# Chemical Speciation of Vanadium(IV/V)/8-Hydroxyquinoline-2-Carboxylic Acid System in Aqueous Solution: A Multitechnique Study

**DOI:** 10.1021/acs.inorgchem.5c05972

**Published:** 2026-03-16

**Authors:** Matteo Marafante, Oluseun Akintola, Vittorio Bariosco, Stefano Bertinetti, Debora Fabbri, Benjamin Kintzel, Winfried Plass, Sofia Gama, Demetrio Milea, Silvia Berto

**Affiliations:** † Dipartimento di Chimica, 9314 Università di Torino, Via P. Giuria 7, 10125 Torino, Italy; ‡ Institut für Anorganische und Analytische Chemie, 9378 Friedrich-Schiller-Universität Jena, Humbolstraße 8, 07743 Jena, Germany; § Department de Química, Universitat Autònoma de Barcelona, E-08193 Bellaterra, Catalonia, Spain; ∥ Centro de Ciências e Tecnologias Nucleares (C2TN), Instituto Superior Técnico, Universidade de Lisboa, Estrada Nacional 10 (km 139.7), 2695-066 Bobadela LRS, Portugal; ⊥ Dipartimento di Scienze Chimiche, Biologiche, Farmaceutiche ed Ambientali (CHIBIOFARAM), 18980 Università degli Studi di Messina, Viale Ferdinando Stagno d’Alcontres, 31, 98166 Messina, Italy

## Abstract

Recent studies have highlighted the biological significance of 8-hydroxyquinoline-2-carboxylic acid (8-HQA) as a metal chelator. In this work, several techniques were applied for the study of the interaction of 8-HQA with vanadium­(IV/V) oxidometal ions at a temperature of *T* = 298.2 K and an ionic strength of *I* = 0.20 mol dm^–3^ in KCl_(aq)_. The redox behavior of the chemical system was defined, and the stability constants of the formed complexes were determined by H^+^-ion selective electrode potentiometry and UV–vis spectrophotometric titrations, while nuclear magnetic resonance (NMR) and electron spin resonance (ESR) spectroscopies and mass spectrometry provided stoichiometric and structural information. The formation of both oxidovanadium­(IV) and dioxidovanadium­(V) ML complexes with 8-HQA was observed in aqueous solution, with both complexes being particularly stable in acidic conditions. The dioxidovanadium­(V) compound is stable even at neutral pH, but dimeric or tetrameric hydrolytic species are predominant under alkaline conditions. In contrast, oxidovanadium­(IV) complexes undergo oxidation as the pH increases. Nevertheless, under strictly anaerobic conditions, the complexation of oxidovanadium­(IV) by 8-HQA can also occur at pH > 6.0. The nature of the oxidovanadium­(IV) and dioxidovanadium­(V) ML complexes, representing the major species formed in solution, was further investigated by DFT calculations.

## Graphical abstract

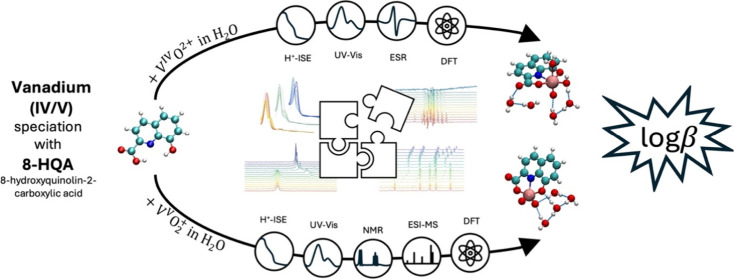

## 1 Introduction

8-hydroxyquinolines (8-HQs) represent a class of compounds characterized by interesting biological activities and pronounced metal-binding properties. The 8-hydroxyquinoline (8-HQ) structure (see Figure 1a) represents a key scaffold for the development of new drug candidates.^1^ In recent years, a large variety of 8-HQ derivatives have been investigated for their potential biomedical applications, such as antifungal, antibacterial, antiproliferative, and anticancer agents.^2,3^ The pharmacological response to 8-HQs in vivo depends on several factors. Multiple studies have highlighted the role of their interaction with metal ions as one of the most relevant aspects to consider, given that the 8-HQs activity is often related to their function as chelating agents or their ability to sequester essential metal ions.^4^ To better understand their potential behavior in vivo, chemical speciation studies and systematic investigations of metal/8-HQ interactions under biologically relevant conditions are crucial.^5,6^ These approaches provide insight into the stability and distribution of metal complexes and offer valuable information on the possible roles and limitations of these compounds as metal-binding agents.

**Figure 1:**
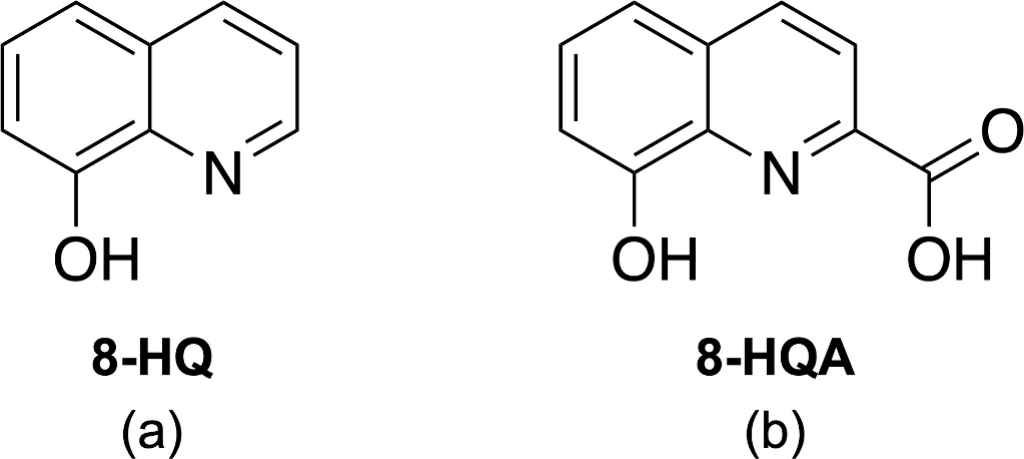
Chemical structures of (a) 8-hydroxyquinoline (8-HQ) and (b) 8-hydroxyquinoline-2-carboxylic acid (8-HQA).

8-hydroxyquinoline-2-carboxylic acid (8-HQA) is an 8-HQ derivative (Figure 1b), naturally occurring in some organisms. In particular, it has been detected in high concentration (0.5–5.0 mmol dm^–3^) in the gut of *Noctuid Larvae* and some other lepidopterans.^7^ Being one of the end metabolites of tryptophan, it seems to have an important role in these organisms as a siderophore in the regulation of the microbiota. In recent years, to elucidate its coordination abilities, 8-HQA has been studied in combination with Fe^2+/3+^, MoO_4_^2–^, Ga^3+^, and other metal ions.^8–12^

Less information is available on the implications of 8-HQA in the human body. Recently, Walczak et al.^13^ reported that 8-HQA presents antiproliferative and antimigratory activity toward colon cancer cell lines, with an observed decrease in cellular DNA synthesis, while no effect was observed on healthy epithelial cells isolated from normal human colon tissue, as well as on the development and viability of zebrafish larvae.

In the past years, 8-HQA was identified as a promising antituberculosis and, more generally, antibacterial agent. Capodagli et al.^14^ investigated the potential of 8-HQA as a selective inhibitor of class II FBA (fructose-1,6-biposphate aldolase), an essential enzyme for various Gram-positive and Gram-negative bacteria, such as *Mycobacterium tuberculosis*, *Escherichia coli*, *Streptococcus pneumoniae*, and *Candida albicans*. Interestingly, 8-HQA showed effective inhibition of class II FBA without inhibiting human and rabbit class I FBAs. Being class II FBAs, a zinc metalloenzyme, while class I FBAs present no metal in the active center, the interaction of 8-HQA with the metal ion seems to have a key role in its activity, together with the structural characteristics of the molecule itself.

As already mentioned, being the activity of 8-HQA (and, more generally, of several 8-HQ derivatives) related to their metal-chelating abilities or their interaction with metal ions in solution,^2^ investigating the chemical speciation and the interactions of 8-HQA with the most relevant metal ions of biological fluids and natural waters appears to be a fundamental aspect to elucidate the features of this naturally occurring compound in both living organisms and environment.^6^

Among the large number of metal ions of interest, particularly those considered relevant in human biochemical processes, vanadium is an essential element for many living organisms.^15^ Being vanadium­(IV) and vanadium­(V) the most representative, relevant, and abundant oxidation states in biological fluids,^16–18^ vanadium (IV/V) and its complexes have been widely studied in the last decades for their implications in human biochemistry and their possible application in medicinal chemistry. Particular attention has been paid to vanadium compounds exploited for their insulin-mimetic effect.^16,19–21^ Furthermore, several vanadium coordination compounds are reported in the literature as potential anticancer, antimicrobial, antibacterial, and antiparasitic agents.^22–27^ This is also the case of oxidovanadium­(IV) complexes of 8-HQ derivatives, which have been studied for their interesting biological activities, presenting anticancer properties.^28,29^ The most relevant biological implications and potential pharmacological applications of vanadium and its compounds have been summarized and reviewed in the past years.^18,30–33^

With all this in mind, the chemical speciation of both oxidovanadium­(IV) (V^IV^O^2+^) and dioxidovanadium­(V) (VO_2_^+^) was investigated in the presence of 8-HQA, pointing out the peculiar features of the considered chemical systems. The present study attempts to provide a meaningful and well-defined framework to understand the coordination behavior of 8-HQA with the two most relevant vanadium oxidation states, oxidovanadium­(IV) (V^IV^O^2+^) and dioxidovanadium­(V) (VO_2_^+^), at *T* = 298.2 ± 0.1 K and *I* = 0.2 mol dm^–3^ KCl_(aq)_. These conditions cannot obviously cover all those that can be encountered under physiologically and/or environmentally relevant scenarios due to the complexity of real systems (characterized by very variable chemical and physical conditions like, e.g., temperature, ionic strength, redox conditions, chemical composition, including competing ions and ligands). However, laboratory studies in well-defined, simplified conditions represent a valuable starting point and the prerequisite for exploring and modeling vanadium (and 8-HQA) behavior in real environments.

## 2 Experimental Section

### 2.1 Chemicals

All the chemicals used were commercially available. 8-hydroxyquinoline-2-carboxylic acid (8-HQA, differently protonated form: (8-hqa)­H_*n*_^2–*n*^), oxidovanadium­(IV) sulfate pentahydrate (VOSO_4_·5H_2_O), sodium metavanadate (NaVO_3_)_,_ potassium permanganate solution, potassium hydrogen phthalate, tris­(hydroxymethyl)­aminomethane (TRIS), ethanol (absolute), potassium chloride, and the concentrated ampules (Tritisol) of potassium hydroxide and hydrochloric acid were purchased from Merck KGaA (Germany), at the highest available purity grade and used without further purification. Ultrapure water (*R* = 18.2 MΩ cm^–1^), produced by a Milli-Q system, was used to prepare all the solutions and samples. 8-HQA solutions were prepared either by weight using an analytical balance by Sartorius (Germany) or by dilution of stock solutions. The minimum known amount of absolute ethanol (always ≤2% v/v) was used to favor the initial solubilization of the ligand in stock solutions. Oxidovanadium­(IV) and dioxidovanadium­(V) stock solutions were prepared by the dissolution of VOSO_4_·5H_2_O and NaVO_3_, respectively. Oxidovanadium­(IV) solutions were standardized against a standard KMnO_4_ solution.^34–37^ While a gravimetric method was exploited to standardize the dioxidovanadium­(V) solutions.^38,39^ Vanadate stock solutions were prepared by dissolving NaVO_3_ in hot water. After cooling to room temperature, the solutions were filtered and evaporated, and then the solid was dried at 383 K to constant weight. In all studies, the ionic strength of sample solutions was adjusted to *I* = 0.2 mol dm^–3^ by means of KCl_(aq)_. KOH_(aq)_ and HCl_(aq)_ solutions were prepared by diluting concentrated ampules and standardized by potassium hydrogen phthalate and TRIS, respectively (dried for at least 24 h at *T* = 383 K prior to usage). Carbonate-free KOH_(aq)_ was prepared weekly using freshly boiled ultrapure water, stored in dark bottles, and protected from atmospheric CO_2_ using soda lime traps.

### 2.2 Potentiometric Measurements

The potentiometric apparatus consisted of a Metrohm 888 Titrando titrator equipped with a combined H^+^-ISE (H^+^-ion selective electrode) Unitrode (Metrohm, model 6.0259.100). Tiamo 2.5 software was used to control the potentiometric experiments and the titration parameters, such as titrant delivery rate, potential stability (±0.2 mV min^–1^), and data acquisition. The titrations were conducted at a constant temperature of *T* = 298.2 ± 0.1 K, under purified N_2_. 25 or 50 cm^3^ of acidified samples (pH ∼ 2, by known amounts of standardized HCl_(aq)_ as detailed below) was titrated using a standardized KOH_(aq)_ solution as titrant (*c*_OH^–^_ = 0.1–0.2 mol dm^–3^). Back titrations were carried out using standardized HCl_(aq)_ solution as titrant (*c*_H^+^_ = 0.1–0.2 mol dm^–3^). Titrated solutions contained different amounts of metal cations (0.25 mmol dm^–3^ ≤ *c*_VO^2+^_ or *c*_VO_2_^+^_ ≤ 1 mmol dm^–3^), 8-HQA (0.5 mmol dm^–3^ ≤ *c*_8‑HQA_ ≤ 1 mmol dm^–3^), and HCl (2 mmol dm^–3^ ≤ *c*_H^+^_ ≤ 10 mmol dm^–3^), in different ratios of oxidovanadium­(IV) or dioxidovanadium­(V) and 8-HQA. The details of each titrated solution are reported in Table S5. In order to minimize oxidation processes due to oxygen in solution, a set of potentiometric titrations was conducted on oxidovanadium­(IV)- and 8-HQA-deaerated solutions. All the solutions used for this set of titrations, as well as the titrated solution, were deaerated and maintained under a constant flux of water-saturated Ar, instead of N_2_. The used electrode was calibrated before each titration for free hydrogen ion concentration, according to the method described by Irving et al.^40^ For each titration, 60–120 points were acquired.

### 2.3 UV–vis Spectrophotometric Measurements

UV–vis absorption spectra were recorded on a Varian Cary 100 Scan (Varian Inc., USA) and Jasco V-550 and Jasco V-750 (Jasco Europe, Italy) UV–vis spectrophotometers. A 200 ≤ λ/nm ≤ 900 spectral window was investigated to study the systems of interest, and quartz cuvettes (Hellma Analytics) with optical path lengths of 1, 10, or 50 mm were selected as a function of the absorbance of the solutions under study. Flow-through cells coupled with a peristaltic pump were used to conduct spectrophotometric titrations.

To obtain absorbance values falling in the linear range of Lambert–Beer’s Law, concentrations of 8-HQA in the range 0.02 mmol dm^–3^ ≤ *c*_8‑HQA_ ≤ 0.1 mmol dm^–3^ were used.

UV–vis spectrophotometric titrations were also conducted to study the interaction between oxidovanadium­(IV) and 8-HQA. Every UV–vis titration included 30–40 test points in a pH range from 2.0 to 11.0. Due to the high difference between the molar absorption coefficient of the aqueous metal ion [VO­(H_2_O)_5_]^2+^ (ε_max_^765^ ∼ 16 mol^–1^ cm^–1^ dm^3^ and ε_max_^635^ ∼ 7 mol^–1^ cm^–1^ dm^3^)^41,42^ and the protonated ligand (8-hqa)­H_2_ (ε_max_^250^ ∼ 40,000 mol^–1^ cm^–1^ dm^3^, see Figure S1), the two families of bands could not be easily observed in the same spectra. To overcome this issue, separate measurements were conducted to investigate only the ligand-related bands, 200 ≤ λ/nm ≤ 500, or the metal-related bands, 500 ≤ λ/nm ≤ 900 with different metal and ligand concentrations in the two cases, to always obtain acceptable absorbance signals.

### 2.4 ESR Measurements

The CW-ESR spectra for the frozen solutions at liquid nitrogen temperatures were recorded in 5 mm quartz tubes using an X-Band ESR-ELEXSYS E580 Spectrometer (Bruker, Germany) equipped with an SHQE resonator (Bruker ER4122SHQE) at a microwave frequency of 9.33 GHz. Spectra were acquired with a modulation amplitude of 0.4 mT and an attenuation of 20 dB. Low temperatures were achieved with a Quartz Finger Dewar insert coupled with a digital temperature control system ER4121VT from Bruker.

Several samples were prepared using different metal-to-ligand ratios, from 1:2 to 1:5, with *c*_8‑HQA_ = 1 mmol dm^–3^. CW-ESR spectra of oxygen-free solutions were also acquired, and Schlenk ESR tubes were used for the preparation of the samples, applying standard Schlenk techniques (under N_2_).^43^

### 2.5 NMR Measurements

^1^H- and ^51^V-NMR spectra were recorded on a 400 MHz Bruker spectrometer (Bruker, USA), using 5 mm NMR tubes. The analyzed samples were prepared in aqueous solution at *I* = 0.2 mol dm^–3^ in KCl_(aq)_. The D_2_O lock was achieved by inserting a small, sealed capillary into the NMR tubes, containing deuterated water and 3-(trimethylsilyl)­propionic-2,2,3,3-d_4_ acid sodium salt (TSP) or neat VOCl_3_, used as a reference to set the δ = 0 ppm for the ^1^H- and ^51^V-NMR spectra, respectively. The signal of water in the ^1^H-NMR spectra was suppressed using a suitable pulse sequence.

To perform pH-dependent NMR experiments, acidic solutions containing both dioxidovanadium­(V) and 8-HQA in the desired metal-to-ligand ratio (specifically, 1:1 or 1:2) were divided into several samples. A precise amount of standardized base (*c*_OH^–^_ = 0.1 mol dm^–3^) was then added to the samples in order to obtain different pH values in the range 2.0 ≤ pH ≤ 11.0. After preparation of the samples, the electrochemical potential of the solutions was measured with a calibrated glass electrode (as described before), and NMR spectra were acquired. ^51^V-NMR spectra were recorded in the −2000 ≤ δ/ppm ≤ 2000 range, though peaks were only observed in the −400 ≤ δ/ppm ≤ −600 range, where peaks of dioxidovanadium­(V) hydrolytic and coordination species are most frequently located. Each experiment series comprises between 20 and 30 independent measurements, at different pH values.

### 2.6 ESI-MS Measurements

Positive- and negative-ion mode ESI-MS (electrospray ionization–mass spectroscopy) spectra were acquired with a Linear Ion Trap Quadrupole LC-MS/MS Mass Spectrometer 3200 QTRAP (AB Sciex Instruments, USA). Several samples at different pH values were prepared using different metal-to-ligand ratios, from 1:1 to 1:2, with *c*_8‑HQA_ = 0.02 mmol dm^–3^. No KCl_(aq)_ was added to avoid the formation of clusters. The pH of the analyzed solutions was adjusted using HCl_(aq)_ (*c*_H^+^_ = 0.2 mol dm^–3^) or KOH_(aq)_ (*c*_OH^–^_ = 0.1 mol dm^–3^). The solutions were injected into the ESI chamber at a flow rate of 10 μL min^–1^. Spectra were recorded in the range 50–400 *m*/*z* and the instrumental conditions were: ion spray voltage ± 3500 V (depending on the polarization mode), source temperature *T* = 573.15 K, curtain gas pressure 20.00 psi, GS1 pressure 20.00 psi, GS2 pressure 20.00 psi, and declustering potential (DP) was varied in the range −100 ≤ DP (eV) ≤ −10 for the negative mode, and 10 ≤ DP (eV) ≤ 100 for the positive mode. MS^2^ spectra were recorded by applying increasing values of collision energy (CE), thanks to the application of a ramp from 5 to 130 eV. All of the mass spectra were acquired by Analyst 1.6.3 software.

### 2.7 Data Processing Software

BSTAC4 software^44^ was used to analyze the potentiometric data in order to determine the stability constants of the investigated species. UV–vis data were processed by HypSpec.^45^ Simulations of the obtained spectra were performed using EasySpin^46^ within the MATLAB environment.^47^ Species distribution diagrams were drawn using PyES software.^48^

### 2.8 DFT Calculations

All calculations presented in this work were performed using the recently released ORCA 6.0.0 version.^49^ Both open- and closed-shell systems were initially optimized at the HF-3c level to identify the starting local minima.^50–52^ The conformational space of the structures was studied by means of the Global Optimization Algorithm (GOAT), newly implemented in the ORCA 6.0.0 version,^53^ at the HF-3c level.^54,55^ To prevent surpassing an excessively large energy barrier between conformers, we only included the water molecules in the “up-hill” process. The maximum energy range in the conformer exploration was set to 25 kJ mol^–1^. All the structures found in the ensemble were then optimized at ωB97X-3c^56^ using the densest grid available (defgrid3) and setting the desired convergence criteria. Solvent effects were considered both explicitly and implicitly at the same time. The former was incorporated by adding a limited number of water molecules (ranging from four to six, depending on the complex) around the metal center, whereas the latter was modeled using a conductor-like polarizable continuum model (CPCM).^57,58^ The final energy refinement was done at the DLPNO-B2PLYP^59^ coupled with the def2-TZVPP basis set, using the RIJCOSX approximation^60^ and the def2-TZVPP/C auxiliary basis set. In the end, the TIGHTPNO setting for the DLPNO was adopted. All the structures and associated energies were minima of the potential energy surface (PES), and thus no imaginary frequencies were found. The computation of the *A*- and *g*-tensors was carried out at the PBE0 level,^61^ the EPR-III basis set^62–64^ was adopted for the H and N atoms, the def2-TZVP for the C and O atoms and the CP­(PPP) basis set for the V atom.^65^ All calculations were performed with the aid of the resolution of identity (RI) approximation,^66,67^ adopting the default options activated by the program. The second-order contribution to the *A*-tensor from spin–orbit coupling was considered with the spin–orbit mean field method [SOMF­(1X)].^68,69^ This combination of method and basis set was selected after a detailed benchmark against the result reported by Lagostina et al.,^70^ on the [VO­(H_2_O)_5_]^2+^ aquoion. Finally, the TD-DFT formalism without the Tamm–Dancoff approximation^71^ was used to compute excitation energies and oscillator strengths.^72,73^ The 20 lowest excited states were considered to compute the vertical excitation energies. ωB97X functional^74,75^ was coupled with def2-tzvp basis set,^65,75^ setting the convergence of the SCF to the tightest.

## 3 Results and Discussion

### 3.1 Acid–Base Properties of Metal Cations and Ligand

To perform accurate chemical speciation studies of a metal–ligand system in aqueous solution, it is fundamental to consider the acid–base equilibria of both the studied cation and ligand in the same temperature, medium, and ionic strength conditions of the work (i.e., *T* = 298.2 K and *I* = 0.2 mol dm^–3^ in KCl_(aq)_).

Oxidovanadium­(IV) (V^IV^O^2+^) and dioxidovanadium­(V) (V^V^O_2_^+^) hydrolysis constants, reported in Tables S1–S3, were calculated in the above conditions by applying an extended Debye–Hückel equation^76^ to literature values from refs 37, 38 and 77 , considering V^IV^O^2+^ and V^V^O_2_^+^, respectively, as component (accounting for ionic medium effect on the basis of the so-called Pure Water approach, see, e.g., ref 48).

The acid–base properties of 8-HQA were extensively studied in these conditions by different techniques.^8–10,78^ The protonation constants used in this work are reported in Table S4, while the spectra of the differently protonated ligand species are shown in Figure S1.

### 3.2 Oxidovanadium­(IV)/8-HQA System

#### 3.2.1 Potentiometric Results

The system was initially investigated in the range 2.0 ≤ pH ≤ 11.0. The titration curves show several equivalence points, indicating the occurrence of different processes in solution. Back-titrations were also conducted, and the titration tracks were compared with the direct-titration when plotting pH vs *eq*OH^–^. Direct- and back-titrations do not fully overlap (Figure S2), suggesting the occurrence of irreversible or kinetically hindered processes in solution. One of the irreversible processes can be the oxidation of oxidovanadium­(IV) to dioxidovanadium­(V) species. Nevertheless, other processes should also be considered, as the sluggish formation of oligomeric oxidovanadium­(IV) hydrolytic species.^79,80^ As such, a feasible data set for the determination of the stability constants of the oxidovanadium­(IV) complexes required that the experimental conditions were chosen to avoid or exclude the occurrence of any of these side processes. For that purpose, several coupled direct- and back-titrations were conducted to elucidate the pH region suitable for the determination of the stability constants of interest, specifically, the pH range in which no precipitation, oxidation, or irreversible processes take place.^81^ From these experiments, it was concluded that irreversible processes become significant between pH ∼ 6 and pH ∼ 7 (Figure S3). Noteworthy, no evident precipitation was observed during the potentiometric measurements.

#### 3.2.2 Spectrophotometric Results

UV–vis absorption spectroscopy was used to monitor 8-HQA-associated bands. These bands are sensitive to changes in the chemical environment, such as protonation or metal complexation, often exhibiting characteristic shifts that reflect alterations in electronic distribution and coordination. In Figure 2a, it can be observed that the complexation of V^IV^O^2+^ by 8-HQA (for pH < 6) leads to a significant bathochromic shift compared to one caused by simple deprotonation of (8-hqa)­H_2_. Interestingly, for pH > 6, the absorption bands of 8-HQA return to behaving as in the simple protonation–deprotonation processes (see Figure 2b). The previously described processes can also be observed at 500 ≤ λ/nm ≤ 900. In this region, the [V^IV^O­(H_2_O)_5_]^2+^ ion usually presents two characteristic absorption bands, centered at λ_max_ ∼ 635 nm and λ_max_ = 760 nm.^82^ In the spectra acquired for the V^IV^O^2+^/8-HQA system, at 3 ≤ pH ≤ 6, we cannot observe the mentioned bands. On the contrary, two broad absorption bands are recorded, with λ_max_ = 600 nm and λ_max_ = 820–880 nm (green colored in Figure 3), compatible with the formation of both V^IV^O­(8-hqa) and [(V^IV^O)_2_(OH)_5_^–^]_*n*_ oligomeric species. The hydrolytic species can be formed at pH > 5; therefore, since the bands are visible for more acidic conditions, it is reasonable suppose the coordination of V^IV^O^2+^ by 8-HQA. At pH > 6, the metal bands significantly change position, shape, and intensity. This effect may result from the formation of V^IV^O­(OH)_2(s)_ and its precipitation, leading to an increase in the background in the visible region (as shown in Figure 3). This is particularly evident for samples containing a metal-to-ligand ratio of 1:1. The precipitation process is not compatible with the processing of the data aiming at the determination of the stability constants of the metal complexes. Therefore, samples in which precipitation was observed were excluded from the calculations. Also for pH > 6, the oxidation of oxidovanadium­(IV) to dioxidovanadium­(V) process becomes significant, revealed by the gradual disappearance of the characteristic oxidovanadium­(IV) absorption bands (see Figure 3), as vanadium­(V) species do not feature any d-d transition that can absorb in the visible region, due to its d^0^ electronic configuration. Although experiments were conducted under an inert atmosphere to limit the level of exposure to O_2_, the oxidation process still occurred, suggesting a marked tendency of oxidovanadium­(IV) to oxidize under these conditions.

**Figure 2:**
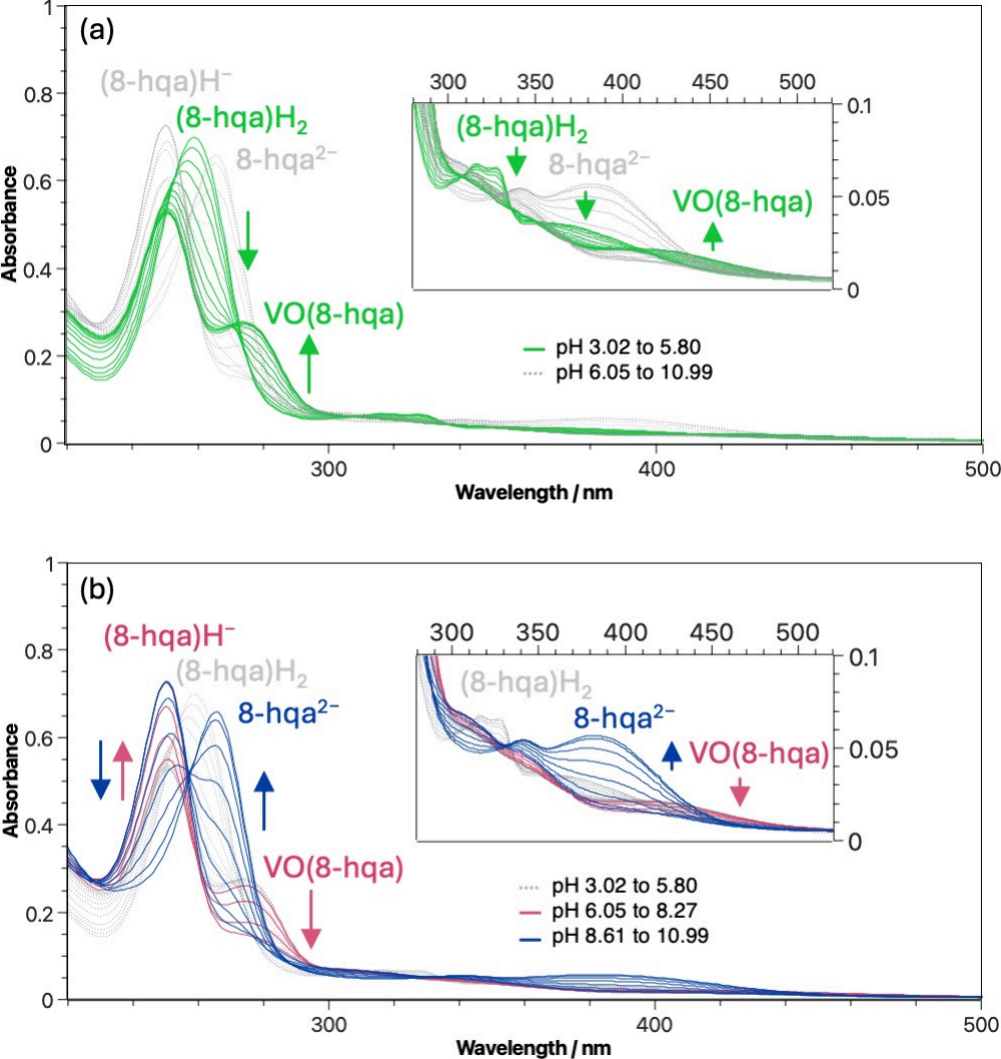
Experimental pH-dependent UV–vis spectra for V^IV^O^2+^/8-HQA aqueous solution (*c*_V^IV^O^2+^_ = 0.01 mmol dm^–3^, *c*_8‑HQA_ = 0.02 mmol dm^–3^, *I* = 0.2 mol dm^–3^ in KCl_(aq)_, *T* = 298.2 K). (a) Green spectra are representative of the V^IV^O­(8-hqa) complex formation and (b) red spectra indicate the decomposition of the V^IV^O­(8-hqa) complex, while blue spectra correspond to the bands of the deprotonated ligand. Optical path length = 10 mm.

**Figure 3:**
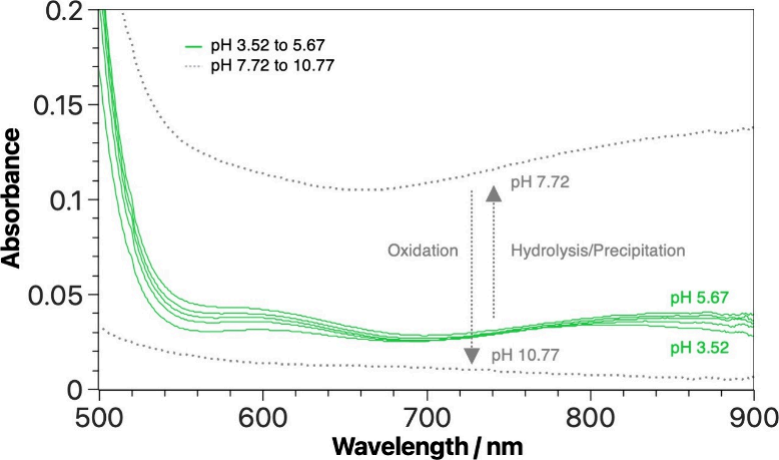
Experimental pH-dependent UV–vis spectra for V^IV^O^2+^/8-HQA aqueous solution (*c*_V^IV^O^2+^_ = 0.5 mmol dm^–3^, *c*_8‑HQA_ = 1.0 mmol dm^–3^, *I* = 0.2 mol dm^–3^ in KCl_(aq)_, *T* = 298.2 K). Green spectra correspond to the stability region for V^IV^O­(8-hqa). Optical path length = 10 mm.

The low solubility of the ligand avoided acquiring visible spectra having absorbance values suitable for stability constants determination; therefore, only spectrophotometric data collected in the UV range were used for the calculation.

#### 3.2.3 Stability Constants of V^IV^O^2+^/8-HQA Complexes

The chemical speciation of oxidovanadium­(IV) in the presence of 8-HQA was investigated by H^+^-ISE potentiometry in the range 2.0 ≤ pH ≤ 6.0. Experimental data analysis evidenced the formation of V^IV^O­(8-hqa) as the main species in this pH range. Nonetheless, other minor species are formed in a limited fraction over the investigated pH range.

At pH < 3.5, a minor fraction (always <20%) of the protonated complex [V^IV^O­(H.8-hqa)]^+^ is formed. On the other hand, at higher pH values, hydrolysis of a coordinated water molecule in V^IV^O­(8-hqa) yields the hydrolytic complex [V^IV^O­(8-hqa)­(OH)]^−^ (as commonly observed for oxidovanadium­(IV) complexes^83,84^ ). In our conditions, the formation of the latter species remains minor since it occurs just prior to extensive oxidation and hydrolysis of oxidovanadium­(IV). Due to their low formation, the stability constants of the protonated and hydrolytic species were determined solely by potentiometric data analysis.

The proposed speciation model and the determined stability constants are reported in Table 1, while an example of a speciation diagram (at *c*_VO^2+^_:*c*_8‑HQA_ = 0.5:1.0 mmol dm^–3^) is shown in Figure 4. As already mentioned, V^IV^O­(8-hqa) is the predominant species at pH ∼ 3.0–6.0, accounting for more than 90% of total oxidovanadium­(IV) at 4.0 < pH < 5.5. The minor species, [V^IV^O­(8-hqa)­(OH)]^−^, only show percentages of ∼20% at pH ∼ 6.0, in the conditions of the diagram in Figure 4. Noteworthy, the stability constants of V^IV^O­(8-hqa) determined both by potentiometry and UV–vis spectrophotometry are in excellent agreement (Table 1). The latter technique allowed the determination of the molar absorption coefficients of this species, showing a maximum at ε_max_^275^ = 2.25 × 10^4^ mol^–1^ cm^–1^ dm^3^.

**Table 1:** Stoichiometry and Stability Constants of the Species Formed in the V^IV^O^2+^/8-HQA Chemical System (*I* = 0.2 mol dm^–3^ in KCl_(aq)_, *T* = 298.2 K)

*p*V^IV^O^2+^ +*q*8-hqa^2–^ +*r*H^+^ ⇄ (V^IV^O^2+^)_*p*_(8-hqa^2–^)_*q*_(H^+^)_*r*_		
*p,q,r*	species^a^	log β^b^
1,1,1	[V^IV^O(H.8-hqa)]^+^	13.2 ± 0.1^c^
1,1,0	V^IV^O(8-hqa)	10.72 ± 0.02^c^
		10.69 ± 0.01^d^
1,1,–1	[V^IV^O(8-hqa)(OH)]^−^	4.04 ± 0.03^c^

a Coordinated water molecules are omitted.

b ±Standard deviation.

c Determined by H^+^-ISE potentiometric titrations.

d Determined by UV–vis spectrophotometric titrations.

**Figure 4:**
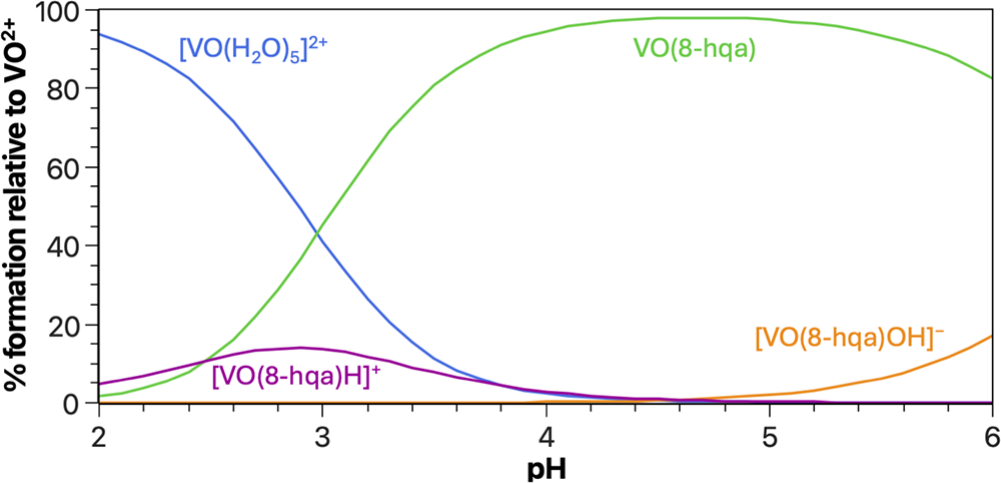
Distribution diagram of V^IV^O^2+^ species in the V^IV^O^2+^/8-HQA system (*c*_V^IV^O^2+^_ = 0.5 mmol dm^–3^, *c*_8‑HQA_ = 1.0 mmol dm^–3^, *I* = 0.2 mol dm^–3^ in KCl_(aq)_, *T* = 298.2 K).

#### 3.2.4 CW-ESR Spectroscopy

A series of pH-dependent ESR spectra was acquired on the oxidovanadium­(IV)/8-HQA system (Figure 5a). At pH < 6, changes in the spectra suggest that complexation occurs, namely, at neutral to basic pH, both a broadening and a decreasing intensity of the spectra are observed. At pH ∼ 8.5, the signal completely disappears, which might be a result of (i) the formation of ESR-silent hydroxido-bridged oxidovanadium­(IV) species,^85^ (ii) the formation of [(V^IV^O)_2_(OH)_5_^–^]_*n*_ oligomeric species, or (iii) the oxidation of oxidovanadium­(IV) to dioxidovanadium­(V).^86^ To check the oxidation hypothesis, pH-dependent ESR experiments were performed in the absence of oxygen (Figure 5b). In these conditions, the characteristic bands of V­(IV) remain present, even at high pH values. This indicates that the absence of signal at pH > 8.5 in aerobic conditions results from the oxidation to dioxidovanadium­(V).

**Figure 5:**
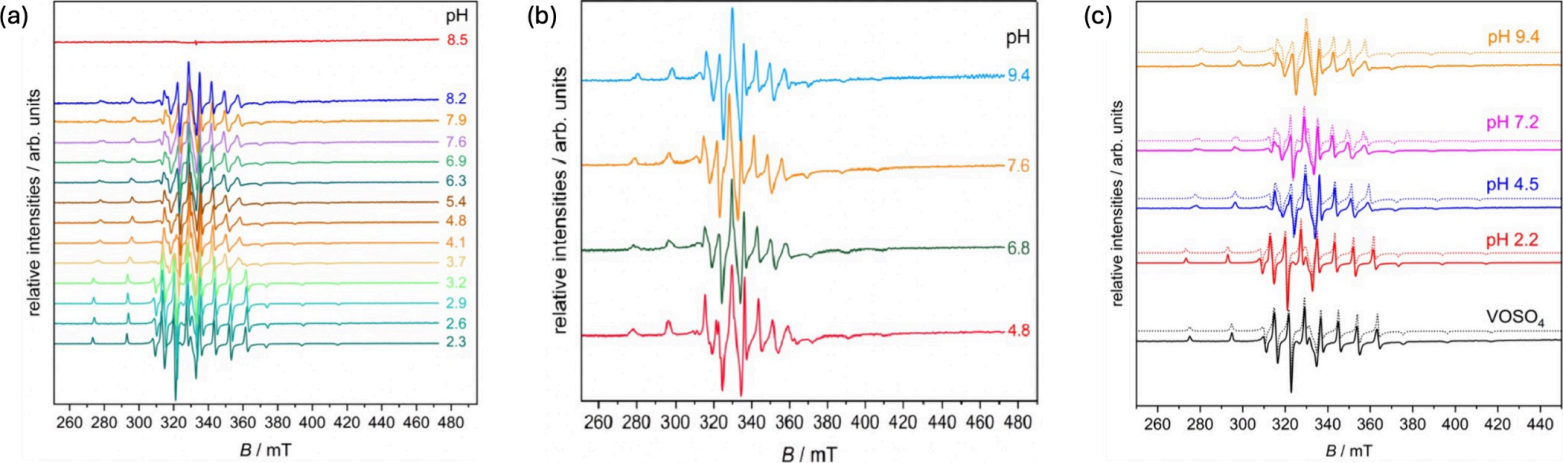
ESR spectra of aqueous solution of V^IV^OSO_4_ and mixtures of V^IV^O^2+^/8-HQA (*c*_V^IV^O^2+^_ = 0.2 mmol dm^–3^, *c*_8‑HQA_ = 0.8 mmol dm^–3^) recorded at (a) *T* ∼ 90 K and pH = 2.2, 4.5, 7.2, and 9.4 with the latter acquired under an inert atmosphere. Dotted lines simulation (see main text and Table 2 for details); (b) pH-dependent experimental spectra acquired at about *T* ∼ 90 K; and (c) experimental spectra fully acquired under anaerobic conditions.

Spectra were simulated to extract the ESR parameters. Experimental and simulated spectra at pH ∼ 2.2, 4.5, 7.2, and 9.4 (the latter spectra acquired under anaerobic conditions) are displayed in Figure 5c, and the corresponding simulated parameters are reported in Table 2. For comparison, spectra of oxidovanadium­(IV) in the absence of 8-HQA were also recorded at pH ∼ 2.2. The ESR parameters obtained at the latter pH, both in the presence and absence of 8-HQA, are consistent with those reported for the aqua complex [V^IV^O­(H_2_O)_5_]^2+^.^87,88^

**Table 2:** ESR Parameters Obtained by the Fitting of Experimental Spectra Presented in Figure 5c^a^

	V^IV^OSO_4_ neat (acid)	pH = 2.2	pH = 4.6	pH = 7.2		pH = 9.4
*g*_⊥_	1.974	1.974	1.974	1.974	1.976	1.976
*g*_∥_	1.928	1.929	1.933	1.933	1.934	1.936
*A*_⊥_ (× 10^–4^ cm^–1^)	69	69	61	60	57	57
*A*_∥_ (× 10^–4^ cm^–1^)	182	182	172	171	166	163
line width (mT)	0.70	0.73	1.0	1.0	1.2	1.1
species	[V^IV^O(H_2_O)_5_]^2+^	[V^IV^O(H_2_O)_5_]^2+^	[V^IV^O(8-hqa)(H_2_O)_3_]	[V^IV^O(8-hqa)(H_2_O)_3_]	[V^IV^O(8-hqa)(OH)(H_2_O)_2_]^−^	[V^IV^O(8-hqa)(OH)(H_2_O)_2_]^−^

a Line width is purely Lorentzian and defined as peak-to-peak.

Upon increasing the pH to 4.5, the spectra change markedly. Simulation of the spectral parameters indicates that the predominant species in solution is monocoordinated complex V^IV^O­(8-hqa). This assignment is supported by the decrease of the *A*_∥_ coupling constants from the initial values at 182 × 10^–4^–172 × 10^–4^ cm^–1^ (Table 2). The experimental A_∥_ value was compared with that calculated using the *additivity rule,* which allows the approximate estimation of *A*_∥_ values as a function of the number and nature of donor groups in the equatorial plane of V^IV^O^2+^. Within this framework, the calculated *A*_∥_ value (*A*_∥_^calc^) of a given V^IV^O^2+^ complex in aqueous solution is obtained by summing the tabulated contributions of different binding moieties.^87,88^

Among the possible V^IV^O­(8-hqa) configurations, two coordination environments are considered most plausible, differing in whether 8-HQA acts as a bidentate or tridentate ligand in the equatorial plane, namely, [V^IV^O­(8-hqa)­(H_2_O)_3_] or [V^IV^O­(8-hqa)­(H_2_O)_2_] (donor sets, *A*_∥_^calc^ values, and schematic structures are reported in Table S6). The experimental *A*_∥_ value for V^IV^O­(8-hqa) (171 – 172 × 10^–4^ cm^–1^) is close to those predicted by the *additivity rule* for both coordination modes, with *A*_∥_^calc^ = 170 × 10^–4^ cm^–1^ for [V^IV^O­(8-hqa)­(H_2_O)_3_] and *A*_∥_^calc^ = 167 × 10^–4^ cm^–1^ for [V^IV^O­(8-hqa)­(H_2_O)_2_]. However, the better agreement for the [V^IV^O­(8-hqa)­(H_2_O)_3_] configuration suggests a bidentate coordination mode of the 8-HQA ligand.

The spectrum recorded at pH ∼ 7.2 exhibits features indicative of the presence of more than one species. Multicomponent simulation reveals contributions from both V^IV^O­(8-hqa) and the oxidovanadium­(IV) hydrolytic species [V^IV^O­(8-hqa)­(OH)]^−^, in agreement with H^+^-ISE potentiometric results. For the latter species, an experimental *A*_∥_ value of 166 × 10^–4^ cm^–1^ was obtained. Application of the *additivity rule* yields *A*_∥_^calc^ values of 163 × 10^–4^ cm^–1^ for [V^IV^O­(8-hqa)­(OH)­(H_2_O)_2_]^−^ and 160 × 10^–4^ cm^–1^ for [V^IV^O­(8-hqa)­(OH)­(H_2_O)]^−^ (Table S6), in good agreement with the experiment. Consistent results were also obtained at pH ∼ 9.4, which yield an experimental *A*_∥_ value of 163 × 10^–4^ cm^–1^, even though at this pH value, the formation of the hydrolytic species [V^IV^O­(OH)_3_]^−^ is not completely negligible. The close agreement between the *A*_∥_^calc^ value for the [V^IV^O­(8-hqa)­(OH)­(H_2_O)_2_]^−^ configuration again supports a bidentate coordination mode of the 8-HQA ligand. Nevertheless, ESR data are insufficient to discriminate unambiguously between bidentate and tridentate coordination.

#### 3.2.5 DFT Calculation

DFT calculations were performed to gain insight into the structure of the V^IV^O­(8-hqa) complex, which represents the most relevant coordination complex in the speciation of the oxidovanadium­(IV)/8-HQA system. According to the literature, 8-HQA may act both as tridentate or bidentate, either via the phenolic oxygen and pyridinic nitrogen or via the carboxylic oxygen and pyridinic nitrogen. However, the latter binding mode is less probable.^8,11,12^ For this reason, two distinct configurations were considered as starting point for the calculations: (i) *tridentate* configuration, with the contribution of the carboxylate group to the coordination of the oxidovanadium­(IV) ion (three donor groups: O^–^, N, COO^–^, structure represented in Figure S6a) and (ii) *bidentate* configuration, where the carboxylate does not bind, and 8-HQA coordinates only via the phenolic oxygen and pyridinic nitrogen (two donor groups: O^–^, N, structure represented in Figure S6b). These configurations were separately optimized through a multistep computational approach, and their energies were calculated and compared. To evaluate the quality of the method, the oxidovanadium­(IV) aquoion [V^IV^O­(H_2_O)_5_]^2+^ was also characterized. Benchmarks from existing publications^70,89–92^ were used as references. Details of the computational approach are provided as the Supporting Information (Paragraph 5.1, Figure S5 and Table S7). Two geometries were optimized for the VO­(8-hqa) complex at the HF-3c level (Figure S6a,b) to establish the starting minimum for conformational exploration. Conformational space exploration using the GOAT algorithm was then applied to those structures.^53^ At the HF-3c level, the *bidentate* configuration is more stable by 27.5 kJ mol^–1^ (estimated energies and bond distances of the conformers are discussed in the Supporting Information, Table S8). However, the single-point energy calculation using the ωB97X-3c functional reverses this trend, suggesting that the *tridentate* configuration is more stable by 90 kJ mol^–1^. The results obtained with ωB97X-3c highlighted the necessity of employing the most suitable density functional to describe the structural effects under investigation to ensure reliable conclusions.

To address this, all conformers for *tridentate* and *bidentate* configurations were reoptimized at ωB97X-3c, yielding global minima with an energy difference of only 0.96 kJ mol^–1^ (global minima structures are reported in Figure 6a,b). A final single-point calculation at the DLPNO-B2PLYP/def2-TZVPP level confirmed these findings, showing negligible energy differences between the two configurations. Bond distances as well as the energy differences between the structures are reported in Table 3.

**Figure 6:**
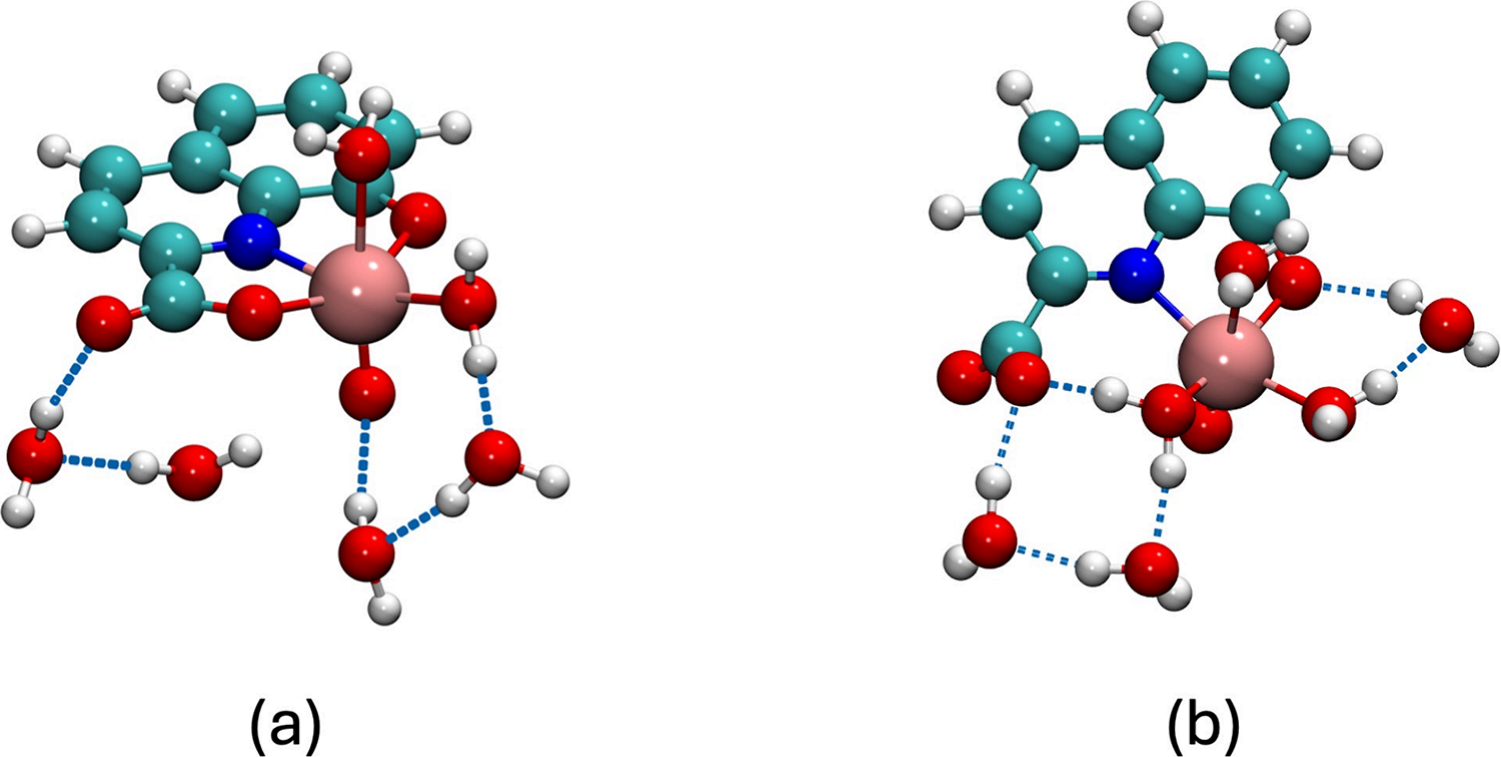
Calculated structures of [V^IV^O­(8-hqa)­(H_2_O)_n_] × (H_2_O)_6–*n*_—global minima obtained after the new ranking at ωB97X-3c level for [V^IV^O­(8-hqa)­(H_2_O)_*n*_] × (H_2_O)_6–*n*_ complex: *tridentate* (a) or *bidentate* (b) configurations. Dashed blue lines represent hydrogen bonds. Color legend: pink, vanadium; red, oxygen; blue, nitrogen; cyan, carbon; and white, hydrogen.

**Table 3:** Distances and Energy Difference Referring to the Structures Reported in Figure 6a,b^a^

	structure	V=O	V–N	V–O (phenol)	V–O^b^
distances (Å)	*tridentate* (Å)	1.58	2.00	1.98	2.05
	*bidentate* (Å)	1.56	2.14	1.93	2.04
energy difference (kJ mol^–1^)	*tridentate–bidentate*	0.96^c^ **/**3.3^d^			

a The unit reported in kJ mol^–1^ is the absolute energy difference between the two structures.

b Distances between the vanadium atom and the oxygen atom of H_2_O in the case of *bidentate conformation* or the oxygen atom of carboxylate in the case of *tridentate binding*.

c Optimized at ωB97X-3c level.

d Single point energy calculated at the DLPNO-B2PLYP/def2-TZVPP//ωB97X-3c level.

Although the conformational barrier between the conformers has not been characterized, the small energy difference between them suggests that both structures can coexist at room temperature, meaning that the carboxylate group can interact with the oxidovanadium­(IV) ion but with the phenolic oxygen and the pyridinic nitrogen undergoing stronger binding.

Based on the DFT results, both UV–vis and ESR spectra of the last global minima obtained at the ωB97X-3c level were simulated. Although the simulated spectra and parameters agree quite well with the experimental data, they do not allow an unambiguous assignment of the observed signals to either of the two configurations considered. Consequently, the DFT study does not enable discrimination between the *tridentate* and *bidentate* configurations. The simulated UV–vis and EPR spectra, together with additional details, are reported in the Supporting Information (Paragraphs 5.4 and 5.5, Figure S9 and Table S9).

#### 3.2.6 NMR Results

For a better understanding of the V^IV^O^2+^/8-HQA system and to further investigate the oxidative process of oxidovanadium­(IV) to dioxidovanadium­(V), several samples of V^IV^O^2+^/8-HQA solutions were prepared, at different pH values, and their ^51^V-NMR spectra were recorded 24 h after their preparation. Some of the obtained spectra are listed in Figure 7. Unlike dioxidovanadium­(V), which is easily detectable by ^51^V-NMR, oxidovanadium­(IV) is not due to its paramagnetic nature. As such, if significant concentrations of dioxidovanadium­(V) species are formed in solution due to the oxidation of oxidovanadium­(IV), ^51^V-NMR signals can be detected. Furthermore, ^51^V-NMR can be exploited to study the different coordination modes of dioxidovanadium­(V) in solution.^39,93–95^

**Figure 7:**
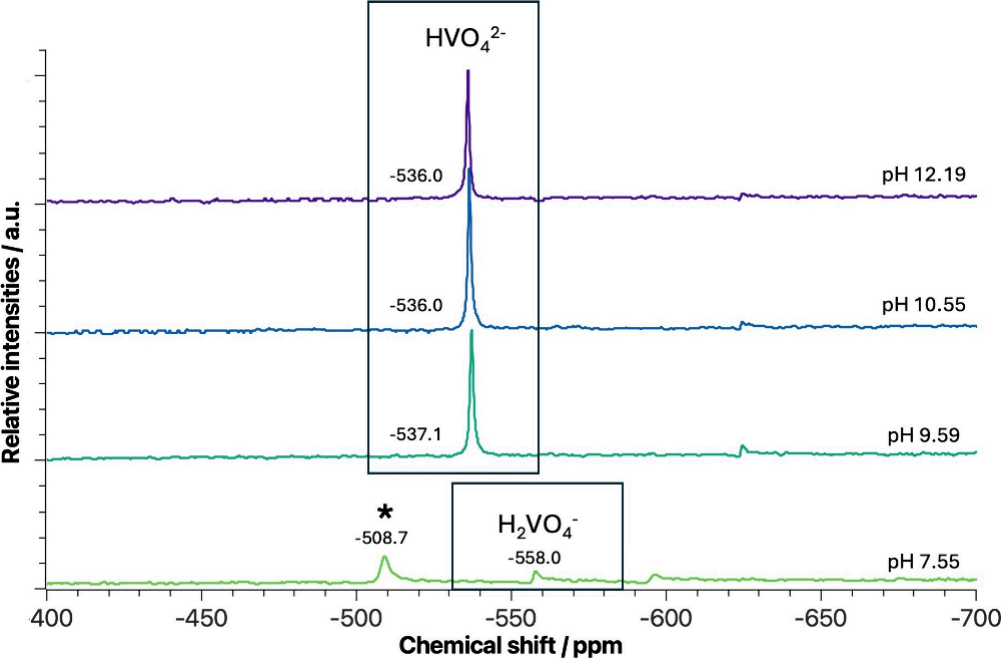
V^IV^O^2+^/8-HQA-aged solution ^51^V-NMR spectra: Experimental pH-dependent ^51^V-NMR spectra for V^IV^O^2+^/8-HQA aqueous solution (*c*_V^IV^O^2+^_ = 0.5 mmol dm^–3^, *c*_8‑HQA_ = 1.0 mmol dm^–3^, *I* = 0.2 mol dm^–3^ in KCl_(aq)_, *T* = 298.2 K). Spectra were recorded 24 h after the preparation of the samples to ensure a settled equilibrium.

In agreement with the above results, under acidic conditions, no ^51^V signals were detected, in the range −2000 ≤ δ/ppm ≤ 2000, confirming the absence or undetectable amounts of vanadium­(V) species in the system. This observation confirms, once again, that the lower oxidation state is stable at acidic pH. Nevertheless, as reported in Figure 7, a non-negligible amount of dioxidovanadium­(V) is generated at pH ≥ 7. The spectra recorded in alkaline solutions show vanadium­(V) signals distributed in the interval −600 ≤ δ/ppm ≤ −500, the chemical shift range characteristic for hydrolytic dioxidovanadium­(V) species.^95,96^ In particular, the signal at δ = −536.0 ppm should correspond to HVO_4_^2–^ ([V^V^O_2_(OH)_3_]^2–^ in V^V^O_2_^+^ formalism) and δ = −558.0 ppm to H_2_V^V^O_4_^–^ ([V^V^O_2_(OH)_2_]^−^ in V^V^O_2_^+^ formalism).^96^ Interestingly, the spectra recorded for the solution at pH = 7.55 show a well-defined peak at δ = −509 ppm (highlighted by an asterisk in Figure 7) that could be attributed to a decavanadate form (V_10_).^96^ Nevertheless, the other decavanadate characteristic peaks, usually located at δ = −425 and δ = −525 ppm, were not detected. Several publications attributed similar results to a ^51^V-NMR signal of dioxidovanadium­(V) complexes.^39,97^ This suggests that the signal at δ = −509 ppm may be related to the formation of a V^V^O_2_^+^/8-HQA species instead of decavanadate clusters, indicative of 8-HQA complexating capabilities toward the higher oxidation state of vanadium, dioxidovanadium­(V) V^V^O_2_^+^.

### 3.3 Dioxidovanadium­(V)/8-HQA Aqueous System

As already mentioned and observed along the study of the system V^IV^O^2+^/8-HQA, the oxidation of the oxidovanadium­(IV) ion leads to the generation of dioxidovanadium­(V) ions that may also be complexed by 8-HQA. To better understand this process, a study of the dioxidovanadium­(V)/8-HQA aqueous system was carried out at 2.0 ≤ pH ≤ 11.0.

Dioxidovanadium­(V) may form a plethora of hydrolytic species with different nuclearities and stability, over the studied pH range (see Table S3).^38^ The equilibria are usually fast, but at 4.0 ≤ pH ≤ 7.0, the slow decomposition of high-nuclearity polyoxidovanadates (namely V_10_) can detrimentally slow down the overall equilibria.^96^ Under the experimental conditions of this work, no significant kinetic hindrance of the equilibrium was observed for the V^V^O_2_^+^/8-HQA system. Indeed, both automatic and *out-of-cell* H^+^-ISE potentiometric titrations with a 24 h equilibration period were performed and compared, showing no significant differences in the potentiometric profiles in this time window.

H^+^-ISE potentiometric titrations and UV–vis spectrophotometry were thus exploited to determine the number, stoichiometry, and stability constants of the species, complemented by ^1^H- and ^51^V-NMR experiments.

#### 3.3.1 Potentiometric Results

Titration curves relative to samples with different metal-to-ligand ratios were recorded and analyzed both qualitatively and quantitatively to determine the nature and stability constants of the formed complexes. The equivalent point observed during the titration is relative to the complete formation of the [V^V^O_2_(8-hqa)]^−^ species. For samples containing a metal-to-ligand ratio of 1:1, the consumed equivalents of titrant (OH^–^) are twice those of 8-HQA. This indicates that, at the equivalent point, the ligand molecules are fully deprotonated, with the pyridinic nitrogen and phenolic oxygen losing their protons, while the carboxylic proton is already released at very acidic pH (see the experimental titration curve reported in Figure S4).

#### 3.3.2 Spectrophotometric Results

UV–vis spectrophotometry was exploited to study the dioxidovanadium­(V) coordination by 8-HQA at different metal-to-ligand ratios. From the pH-dependent spectra reported (Figure 8), we can observe that, in acidic conditions, the coordination of V^V^O_2_^+^ results in a bathochromic shift to a λ_max_ = 273 nm of the free ligand’s bands, which are usually located at 250 ≤ λ/nm ≤ 265 (Figure S1). For the formed [V^V^O_2_(8-hqa)]^−^ species, molar absorption coefficients were estimated with the characteristic maximum being ε_max_^273^ = 3.41 × 10^4^ mol^–1^ cm^–1^ dm^3^. At pH > 6 this band decreases, suggesting the formation and predominance of dioxidovanadium­(V) hydrolytic species in solution.

**Figure 8:**
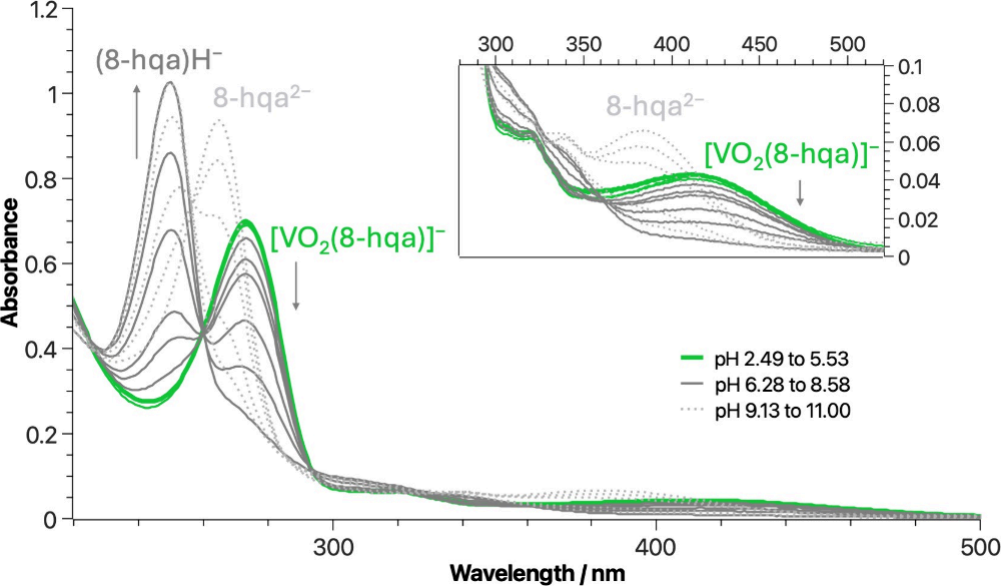
Experimental pH-dependent UV–vis spectra for V^V^O_2_^+^/8-HQA aqueous solution system (*c*_V^V^O_2_^+^_ = 0.02 mmol dm^–3^, *c*_8‑HQA_ = 0.02 mmol dm^–3^, *I* = 0.2 mol dm^–3^ in KCl_(aq)_, *T* = 298.2 K): the spectra highlighted in green are registered in the pH range in which the [VO_2_(8-hqa)]^−^ complex is present. Optical path length = 10 mm.

#### 3.3.3 Complex Stability Constants of V^V^O_2_^+^/8-HQA

The stability constant of the [V^V^O_2_(8-hqa)]^−^ species was determined by both H^+^-ISE potentiometry and UV–vis spectrophotometry. Obtained values, reported in Table 4, show quite a low uncertainty and are in excellent agreement with each other. As shown in the speciation diagram of Figure 9, the [V^V^O_2_(8-hqa)]^−^ complex is formed in very high percentages (90–100% relative to V^V^O_2_^+^) at 2 ≤ pH ≤ 7. Even at different *c*_V^V^O_2_^+^_:*c*_8‑HQA_ ratios, this species is still dominant at pH ≤ 7 (formation percentages >70% at *c*_V^V^O_2_^+^_ = *c*_8‑HQA_ = 0.5 mmol dm^–3^). At pH > 7, V^V^O_2_^+^ hydrolytic species predominate over 8-HQA complexation.

**Table 4:** V^V^O_2_^+^/8-HQA Stability Constants: Stoichiometry and Stability Constants of the Species Refined for the V^V^O_2_^+^/8-HQA Chemical Systems^a^

*p*V^V^O_2_^+^+*q*8-hqa^2–^ +*r*H^+^ ⇄ (V^V^O_2_^+^)_*p*_(8-hqa^2–^)_*q*_(H^+^)_*r*_		
*p,q,r*	species^b^	log β^c^
1,1,0	[V^V^O_2_(8-hqa)]^–^	13.88 ± 0.03^d^
		13.86 ± 0.01^e^

a *I* = 0.2 mol dm^–3^ in KCl_(aq)_, *T* = 298.2 K.

b Coordinated water molecules are omitted.

c ±Standard deviation.

d Determined by H^+^-ISE potentiometric titrations.

e Determined by UV–vis spectrophotometric titrations.

**Figure 9:**
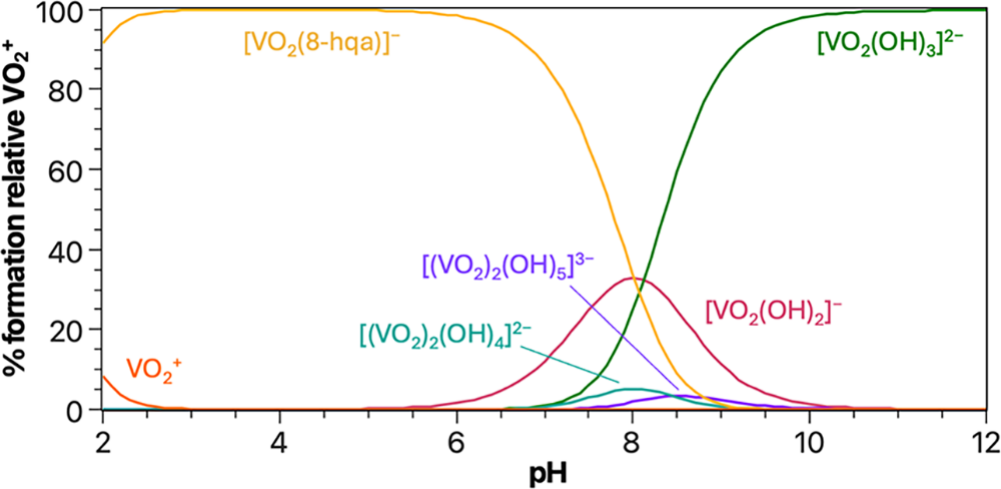
Distribution diagram of V^V^O_2_^+^ species in the V^V^O_2_^+^/8-HQA system (*c*_V^V^O_2_^+^_ = 0.5 mmol dm^–3^, *c*_8‑HQA_ = 1.0 mmol dm^–3^, *I* = 0.2 mol dm^–3^ in KCl_(aq)_, *T* = 298.2 K).

#### 3.3.4 NMR Results

NMR spectroscopy was also exploited to characterize the V^V^O_2_^+^/8-HQA interactions in aqueous solution. Figure 10 shows the ^1^H NMR spectra acquired at different pH levels for the V^V^O_2_^+^/8-HQA system with *c*_V^V^O_2_^+^_:*c*_8‑HQA_ = 1:2 (see also Figure S12). In these conditions, and when compared with the chemical shifts of the ^1^H NMR signals of 8-HQA (reported by Baryłka et al.^78^) two populations of signals are observed at pH < 8.0, corresponding to bound and unbound 8-HQA.^76^ It is possible to assign the less intense group of peaks to uncomplexed 8-HQA (i.e., H_b_, H_c_, H_d_, H_e_, and H_f_), and the other group (i.e., H_b_*, H_c_*, H_d_*, H_e_*, and H_f_*) to a dioxidovanadium­(V) complex (i.e., [V^V^O_2_(8-hqa)]^−^).

**Figure 10:**
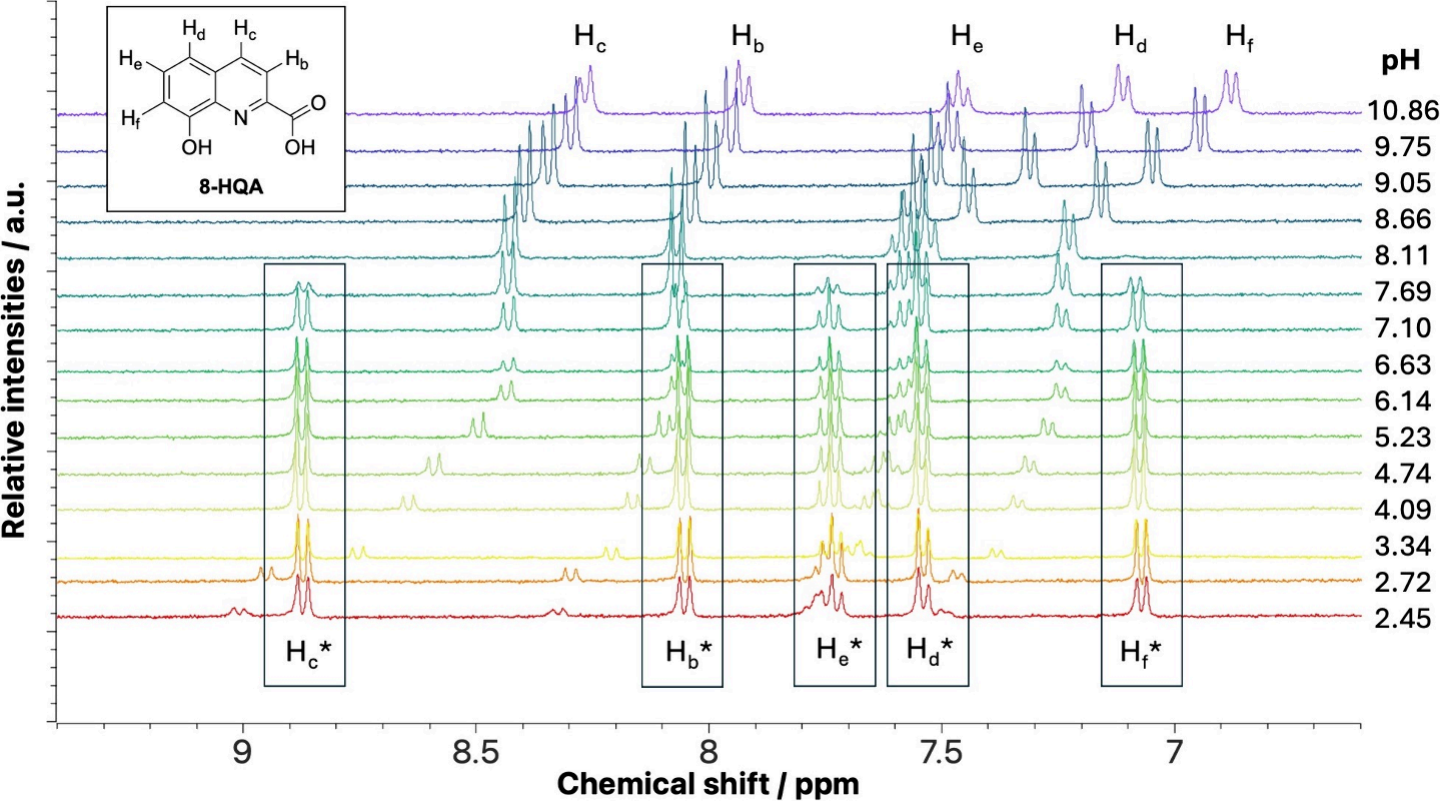
Experimental pH-dependent ^1^H NMR spectra for V^V^O_2_^+^/8-HQA aqueous solution (*c*_V^V^O_2_^+^_ = 0.5 mmol dm^–3^, *c*_8‑HQA_ = 1 mmol dm^–3^, *I* = 0.2 mol dm^–3^ in KCl_(aq)_, *T* = 298.2 K). Spectra were recorded 24 h after the preparation of the samples to ensure a settled equilibrium.

Noteworthy, by ^1^H NMR, we can observe the formation of the complex at very acidic pH, being present in appreciable concentrations up to pH ∼ 8.0. At pH < 7, the ^1^H NMR signals do not undergo any shift, proving that the complex species, formed at acidic pH, remains stable and does not change its nature over a wide pH range, while their intensities decrease at pH > 7.0, in favor of peaks of uncomplexed 8-HQA species. Not surprisingly, in experimental conditions of the metal-to-ligand 1:1 ratio, only the signals of the coordinated 8-HQA are detectable, suggesting that 8-HQA is completely bound to the dioxidovanadium­(V) ion.

The analysis of pH-dependent ^51^V-NMR spectra (see, e.g., Figure 11) at different *c*_8‑HQA_: *c*_V^V^O_2_^+^_ ratios confirms these observations.

**Figure 11:**
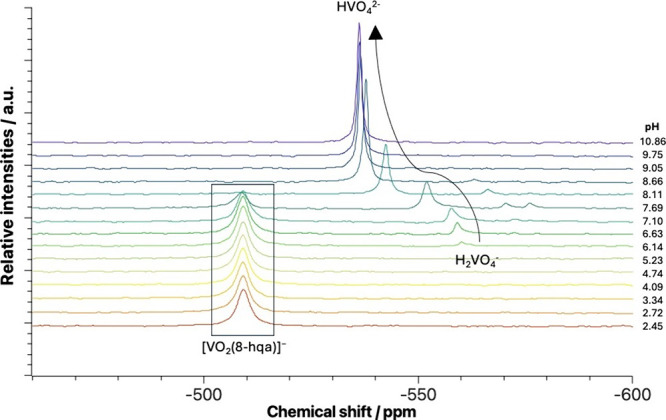
Experimental pH-dependent ^51^V-NMR spectra for V^V^O_2_^+^/8-HQA aqueous solution (*c*_V^V^O_2_^+^_ = 0.5 mmol dm^–3^, *c*_8‑HQA_ = 0.5 mmol dm^–3^, *I* = 0.2 mol dm^–3^ in KCl_(aq)_, *T* = 298.2 K). Spectra were recorded 24 h after the preparation of the samples to ensure a settled equilibrium.

At pH < 6, only one peak appears at δ ∼ −509.0 ppm that can be assigned to the [V^V^O_2_(8-hqa)]^−^ species. At neutral pH, the intensity of this peak starts to decrease, in favor of typical peaks of dioxidovanadium­(V) hydrolytic species.^94^ Noteworthy, the signal of the complex species is localized in the range where one of the three characteristic decavanadate V_10_ peaks usually occurs.^95–98^ Nevertheless, no evidence of decavanadate cluster formation was found, since its typical peaks at δ ∼ −425 and δ ∼ −530 ppm are absent.

Comparing the spectra of 1:1 or 1:2 metal-to-ligand mixtures, acquired in the [V^V^O_2_(8-hqa)]^−^ stability region, shows that no relevant differences in the distributions of the ^51^V-NMR peaks are observed. We can then conclude that the [V^V^O_2_(8-hqa)]^−^ complex seems to be the predominant species, also in the presence of ligand excess.

#### 3.3.5 ESI-MS Results

In combination with other techniques [9], mass spectrometry proved useful even in chemical speciation studies of vanadium complexes.^99^

ESI-MS experiments were conducted on V^V^O_2_^+^/8-HQA aqueous solutions at pH ∼ 5 and a metal-to-ligand ratio of 1:1, where, according to the speciation diagram (Figure 9), the maximum formation of the [V^V^O_2_(8-hqa)]^−^ complex is expected.

The ESI-MS(−) spectrum of free 8-HQA (Figure 12a) highlights the presence of the monoprotonated form HL^–^ (*m*/*z* 188), in which it is hypothesized that the phenolic oxygen remains protonated, while the pyridinic nitrogen and the carboxylic acid group are deprotonated. The only fragmentation pattern observed under the experimental conditions is the loss of the carboxylic acid group as carbon dioxide (Δ*m* = 44 u), generating the *m*/*z* 144 signal observed in both MS and MS^2^ spectra.

**Figure 12:**
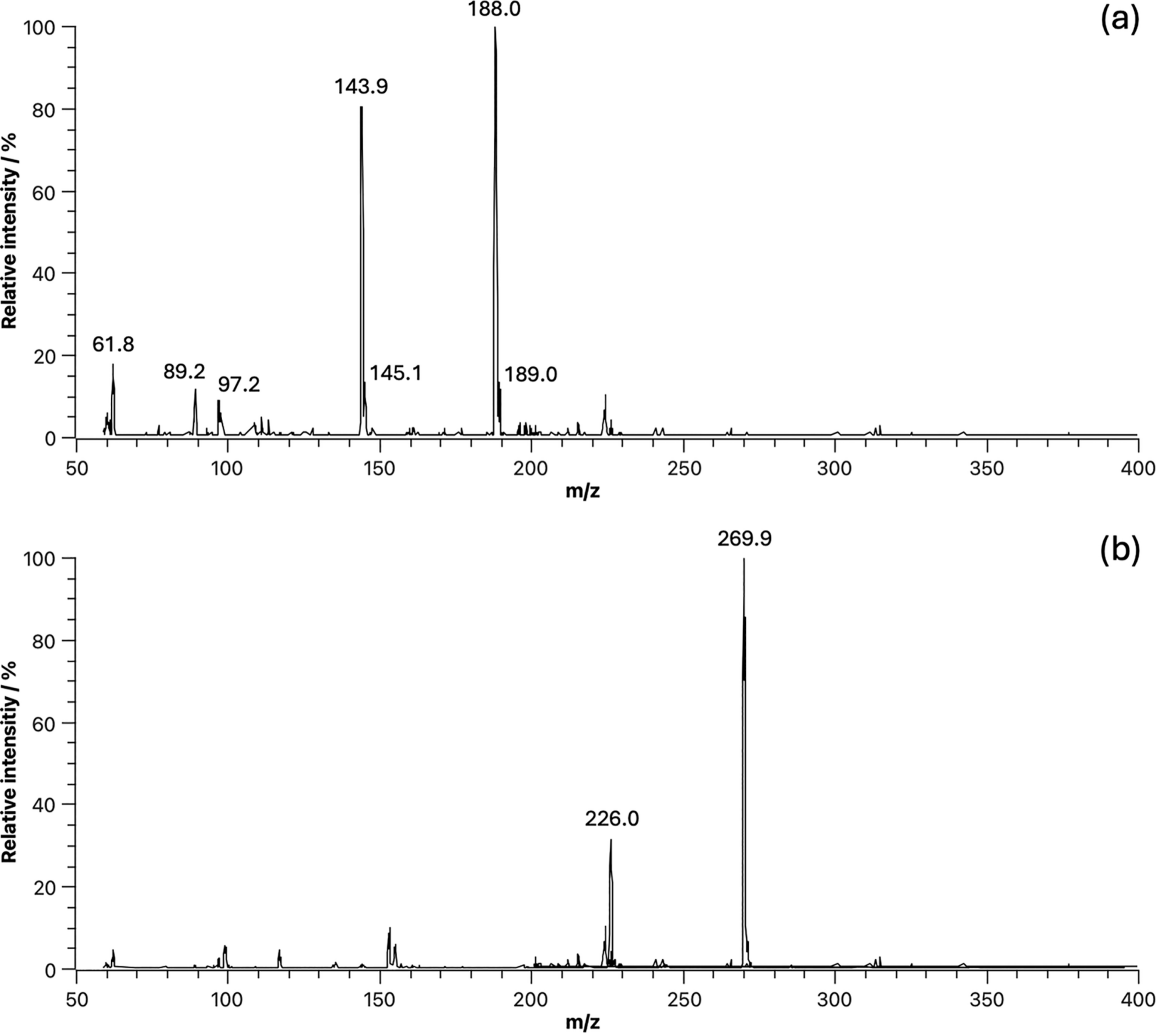
ESI-MS spectra: (a) ESI-MS(−) spectrum for an 8-HQA aqueous solution at pH 5 (*c*_8‑HQA_ = 0.02 mmol dm^–3^); (b) ESI-MS(−) spectrum for V^V^O_2_^+^/8-HQA aqueous solution at pH 5 (*c*_V^V^O_2_^+^_ = 0.02 mmol dm^–3^, *c*_8‑HQA_ = 0.02 mmol dm^–3^).

In ESI-MS­(+), the molecular peak corresponds to the (8-hqa)­H_3_^+^ species with *m*/*z* = 190 (Figure S13). In this form, the phenol, the pyridine, and the carboxylic acid group are all protonated (C_10_H_8_NO_3_^+^, *m*/*z* 190.2).

The spectrum of the V^V^O_2_^+^/8-HQA solution acquired in negative mode (Figure 12b) shows an intense molecular peak at *m*/*z* 270, consistent with the presence of the [V^V^O_2_(8-hqa)]^−^ species (VC_10_H_5_NO_5_^–^, *m*/*z* 269.9). Figure S14 shows the ESI-MS^2^ spectra of the *m*/*z* 270 precursor ion. The fragmentation of the [VO_2_(8-hqa)]^−^ species was studied by varying the CE through a ramp from −130 to −5 eV (step = 5 eV). The only relevant fragment is the peak at *m*/*z* 226, corresponding to the loss of 44 u, which is compatible with the removal of CO_2_ from the carboxylate moiety. The preferential loss of CO_2_, rather than the disruption of the complex, indicates that the coordination of dioxidovanadium­(V) by the ligand is moderately strong.

Differently to ESI-MS(−), in which the [V^V^O_2_(8-hqa)]^−^ complex was observed, in ESI-MS­(+), no significant signals were detected for the V^V^O_2_^+^/8-HQA solution at pH 5 (Figure S15). The lack of any relevant signal supports the hypothesis of chelation by the 8-HQA: in fact, in the case of the absence of coordination, the functional groups of 8-HQA (that usually act as metal binders) would be protonated, giving a signal in the positive-mode mass spectra (as in Figure S13). The absence of the characteristic H_3_L^+^ signal at *m*/*z* 190 clearly indicates that the ligand is involved in coordination with the V^V^O_2_^+^ ion.

Thus, the registered mass spectra suggest that the [V^V^O_2_(8-hqa)]^−^ species (VC_10_H_5_NO_5_^–^, *m*/*z* 269.9) represent the major species present in solution, supporting the other experimental results and agreeing with the distribution diagram reported in Figure 9.

#### 3.3.6 DFT Calculations

Adopting the computational methodology established for the [V^IV^O­(8-hqa)] complex, the conformational space of the [V^V^O_2_(8-hqa)]^−^ structure was investigated. To account for solvation effects, four water molecules were considered. As described for the oxidovanadium­(IV)/8-HQA system, also in this case two distinct configurations were considered: (i) the *tridentat*e configuration, where the carboxylic group coordinates the V^V^O_2_^+^ center (donor groups: O^–^, N, and COO^–^) and (ii) the *bidentate* configuration, in which a water molecule occupies the corresponding coordination site (donor groups: O^–^ and N).

A conformational space exploration conducted by using the GOAT code yielded 38 structures for the *tridentat*e configuration and 21 structures for the *bidentat*e configuration within a 25 kJ mol^–1^ energy window, including the respective global minima. All identified geometries were subsequently reoptimized and ranked at the ωB97X-3c level: the final global minima are depicted in Figure 13a,b. Different from the V^IV^O­(8-hqa) complex, quantum mechanical calculations indicate a preference of the [V^V^O_2_(8-hqa)]^−^ complex for the *tridentat*e configuration, which resulted in ∼30 kJ mol^–1^ more stable than the *bidentat*e counterpart (Table 5).

**Figure 13:**
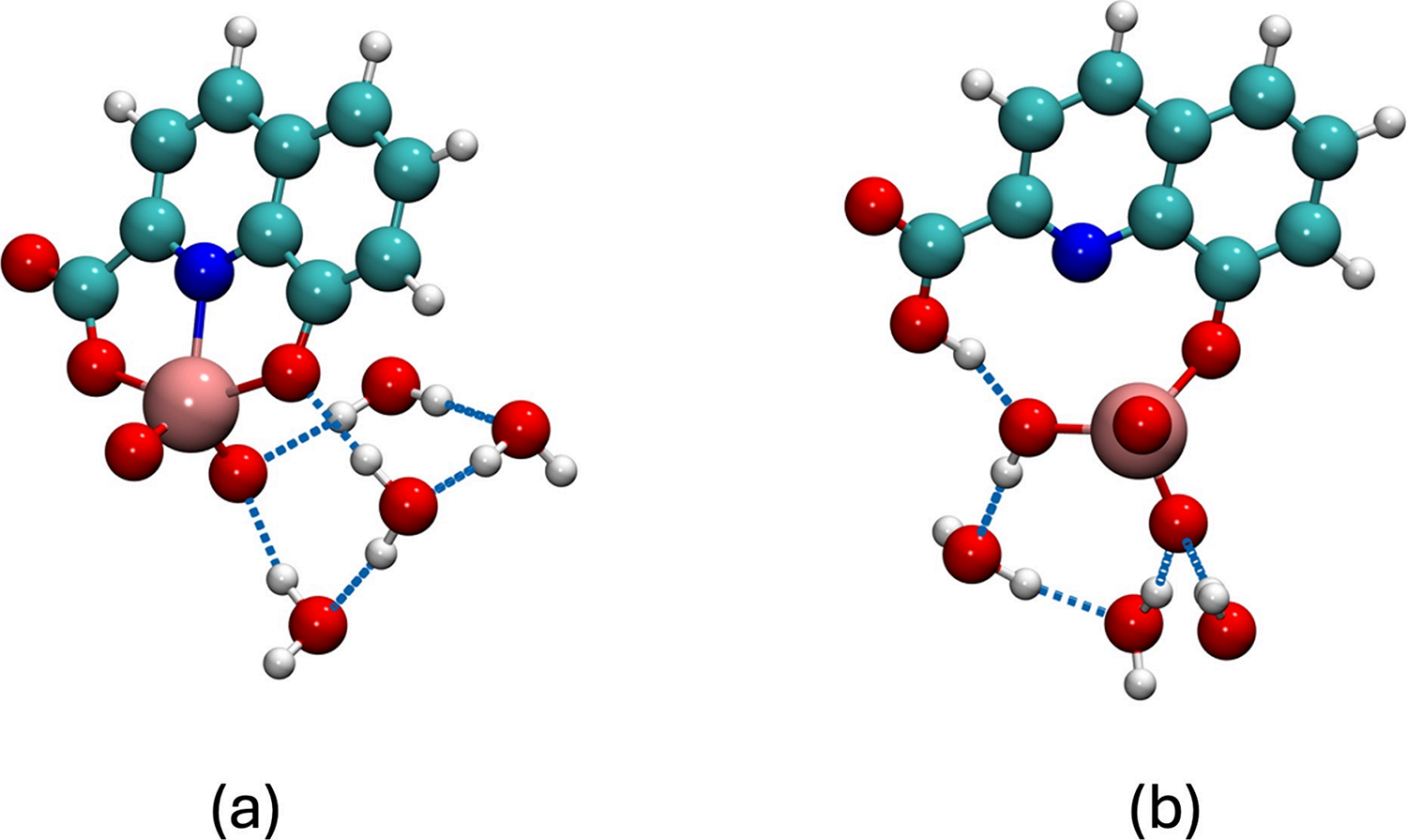
Global minima obtained after the new ranking at ωB97X-3c level for [V^V^O_2_(8-hqa)­(H_2_O)_*n*_]^−^ × (H_2_O)_4–*n*_: *tridentate* (a) or *bidentate* (b) configurations. Dashed blue lines represent hydrogen bonds. Color legend: pink, vanadium; red, oxygen; blue, nitrogen; cyan, carbon; and white, hydrogen.

**Table 5:** Distances and Energy Difference Referring to the Structures Reported in Figure 13a,b^a^

	structure	V=O_1_	V=O_2_	V–N	V–O (phenol)	V–O^b^
distances (Å)	*tridentate* (Å)	1.61	1.58	2.08	1.99	2.01
	*bidentate* (Å)	1.62	1.59	2.91	1.85	1.81
energy difference (kJ mol^–1^)	tridentate–bidentate	–29.6^c^				

a The unit reported in kJ mol^–1^ is the absolute energy difference between the two structures.

b Distances between the vanadium atom and the oxygen atom of H_2_O, in the case of *bidentate* configuration, or the oxygen atom of carboxylate, in the case of *tridentate* configuration.

c Reoptimization energy calculated at ωB97X-3c level.

This energy difference can be attributed to notable differences in the coordination. In particular, the vanadium–nitrogen bond length in the *bidentat*e configuration is approximately 1 Å longer than in the *tridentat*e one. Furthermore, the *bidentat*e configuration undergoes proton transfer from the water molecule coordinating the V^V^O_2_^+^ ion to the oxygen atom of the carboxylate moiety (see Figure 13b). This can be rationalized with the fact that the dioxidovanadium­(V) group shows a higher electrophilic behavior with respect to the oxidovanadium­(IV). This, in turn, means that the oxygen atom of the water molecule chelating the metal center can efficiently delocalize part of its electrons, thus increasing the acidity of the proton.

## 4 Conclusions

The chemical speciation in an aqueous solution of vanadium­(IV) and vanadium­(V) oxidometal ions with 8-HQA was studied. Oxidovanadium­(IV) is coordinated by 8-HQA at acidic pH, forming V^IV^O­(8-hqa) and, in smaller amounts, other minor species as [V^IV^O­(H.8-hqa)]^+^ and [V^IV^O­(8-hqa)­(OH)]^−^. Reliable thermodynamic characterization can be performed only for pH < 6, as oxidation and hydrolysis at higher pH hamper a more accurate identification of other species. V^IV^O­(8-hqa) species is stable at pH < 6 for all the investigated metal-to-ligand ratios, ranging between 1:1 and 1:5. At 5 < pH < 6, [V^IV^O­(8-hqa)­(OH)]^−^ species may form in relatively low formation percentages (∼20%) in the experimental conditions of this work. At a higher pH, oxidovanadium­(IV) is rapidly oxidized. To achieve acceptable redox stability of oxidovanadium­(IV) compounds at neutral or alkaline pH, oxygen must be strictly excluded. Under anaerobic conditions, the presence of oxidovanadium­(IV) complex species was observed by ESR spectroscopy up to pH ∼ 9.0. The stability constants of the complexes were determined by H^+^-ISE potentiometry and UV–vis spectrophotometry. ^51^V-NMR spectra acquired on aged oxidovanadium­(IV)/8-HQA samples at neutral pH confirmed the oxidation of oxidovanadium­(IV) to dioxidovanadium­(V) and showed that dioxidovanadium­(V) is complexed by 8-HQA. The interactions between dioxidovanadium­(V) and 8-HQA were then investigated at 2.0 ≤ pH ≤ 11.0. The [V^V^O_2_(8-hqa)]^−^ species is already formed at acidic pH and is present in solution up to pH ∼ 8, where dioxidovanadium­(V) forms dimeric or tetrameric hydrolytic species together with the most abundant monovanadates.

To summarize, 8-HQA shows binding ability for both oxidovanadium­(IV) and dioxidovanadium­(V) cations in acidic and mild-acidic conditions. In particular, 8-HQA possesses strong chelating abilities toward dioxidovanadium­(V) in really acidic solutions. For oxidovanadium­(IV), chelation becomes relevant only at pH > 3. While oxidovanadium­(IV) is rapidly oxidized at neutral pH values, the dioxidovanadium­(V) complex shows moderate stability still at pH ∼ 7.0.

DFT calculations indicate that 8-HQA can act as either a bidentate or tridentate toward oxidovanadium­(IV), while the latter binding modes seem to be preferred in the case of dioxidovanadium­(V).

Overall, this work establishes a comprehensive description of the oxidovanadium­(IV)/8-HQA and dioxidovanadium­(V)/8-HQA aqueous systems, enabling the prediction of solution speciation as a function of pH, oxygen availability, and total concentration. The provided data enrich the understanding of the behavior and biological relevance of vanadium­(IV/V)/8-HQA complexes and contribute to broader knowledge on 8-HQ-based compounds.

## Supplementary Material



## Notes

Oxidovanadium­(IV) and dioxidovanadium­(V) hydrolytic constants, 8-hydroxyquinoline-2-carboxylic acid protonation constants, and UV–vis spectra; potentiometric titrations: solution composition and titration curves; ESR parameters of oxidovanadium­(IV)/8-hydroxyquinoline-2-carboxylic acid complexes predicted by the additivity relationship; details on DFT calculations; peak position of V^V^O_2_^+^/8-HQA ^1^H NMR spectra as a function of pH; and ESI-MS and ESI-MS^2^ spectra (PDF)
