# Supplementary material for: Chemical Speciation of Vanadium(IV/V)/8-Hydroxyquinoline-2-Carboxylic Acid System in Aqueous Solution: A Multitechnique Study
Source: Inorg Chem. 2026 Mar 16;65(12):6657–71. doi: 10.1021/acs.inorgchem.5c05972 (PMC13040529; doi:10.1021/acs.inorgchem.5c05972)
Supplement: Supplementary file 1 [file ic5c05972_si_001.pdf]

Supporting Information

Chemical speciation of vanadium(IV/V) / 8-hydroxyquinoline-2-carboxylic acid system in aqueous solution: a multi-technique study

Matteo Marafante[a], Oluseun Akintola[b], Vittorio Barioscoa[a,c], Stefano Bertinetti[a], Debora Fabbri[a], Benjamin Kintzel[b], Winfried Plass[b], Sofia Gama[d*], Demetrio Milea[e], Silvia Berto[a*]

[a]*Dipartimento di Chimica, Università di Torino, Via P. Giuria 7, 10125, Torino, Italy*

[b]*Institut für Anorganische und Analytische Chemie, Friedrich-Schiller-Universität Jena, Humbolstraße 8, 07743, Jena, Germany*

[c]*Department de Química, Universitat Autònoma de Barcelona, E-08193 Bellaterra, Catalonia, Spain*

[d]*Centro de Ciências e Tecnologias Nucleares (C²TN), Instituto Superior Técnico, Universidade de Lisboa, Estrada Nacional 10 (km 139.7), 2695-066, Bobadela LRS, Portugal*

[e]*Dipartimento di Scienze Chimiche, Biologiche, Farmaceutiche ed Ambientali (CHIBIOFARAM), Università degli Studi di Messina, Viale Ferdinando Stagno d'Alcontres, 31, 98166 Messina, Italy*

* Corresponding authors:

Silvia Berto

silvia.berto@unito.it

Sofia Gama

sofia.gama@ctn.tecnico.ulisboa.pt

Index

1. **Stability constants** **3**
2. **UV-vis** **7**
3. **Potentiometry** **8**
4. **ESR** **11**
5. **DFT calculations** **12**
6. **NMR** **23**
7. **ESI-MS** **24**

References **25**

1. Stability constants

1.1 Oxidovanadium(IV) ($V^{IV}O^{2+}$) hydrolytic constants

Table S1. Oxidovanadium(IV) hydrolytic stability constants (log β).

$pV^{IV}O^{2+} + rH^+ \rightleftarrows (V^{IV}O^{2+})_p (H^+)_r$		
p,r	species	log β^c
1,-1	$[V^{IV}O(OH)]^+$	-5.83
1,-3	$[V^{IV}O(OH)_3]^-$	-18.2
2,-2	$[(V^{IV}O)_2(OH)_2]^{2+}$	-6.99
2,-5	$[(V^{IV}O)_2(OH)_5]^-$	-21.94

[a] log β at $I = 0.20$ mol·dm^{-3} and $T = 298.2$ K adapted from ref. [1,2]

1.2 Dioxidovanadium(V) ($V^VO_2^+$) hydrolysis constants

Hydrolysis of vanadium(V) is often reported in literature as acid-base equilibria of dihydrogenvanadate ion ($H_2V^VO_4^-$)[3–9], that is the conjugated base of vanadic acid $H_3V^VO_4$, and acts as an ampholytic ion. The formation of the differently protonated or hydrolytic species of $H_2V^VO_4^-$ can be described by a chemical model in which the components are $H_2V^VO_4^-$ and H^+ (see Equation S1). All equilibria considered are reported in Table S2. The hydrolytic constants $\log\beta_{p,r}^{H_2V^VO_4^-}$ extracted from Elvingson *et al.* [3], for $I = 0.15$ mol·dm^{-3} and $T = 298.2$ K, were calculated at $I = 0.20$ mol·dm^{-3} and $T = 298.2$ K by means of an Extended Debye-Hückel Equation (EDH).[10,11]

$$\log\beta_{p,r}^{H_2V^VO_4^-} \qquad p\, H_2V^VO_4^- + rH^+ \rightleftarrows (H_2V^VO_4^-)_p(H^+)_r \qquad \text{(S1)}$$

Hydrolytic species of vanadium(V) can also be described from the equilibria relating the dioxidovanadium(V), $V^VO_2^+$. In this case the components of the chemical model are $V^VO_2^+$ and H^+, as described by Equation S2. The hydrolytic constants that refer to this formalism, $\log\beta_{p,r}^{V^VO_2^+}$, can be found in literature [12,13], or can be derived from $\log\beta_{p,r}^{H_2V^VO_4^-}$ values.

$$\log\beta_{p,r}^{V^VO_2^+} \qquad pV^VO_2^+ + rH^+ \rightleftarrows (V^VO_2^+)_p(H^+)_r \qquad \text{(S2)}$$

$V^VO_2^+$ represents the most stable vanadium(V) form in strong acidic conditions and it is the doubly protonated form of $H_2V^VO_4^-$, as reported in Equation S3.

$V^VO_2^+$ hydrolytic model can be easily derived from $H_2V^VO_4^-$ hydrolytic model and vice versa. As already mentioned, Equation S3 defines the formation of $V^VO_2^+$ from $H_2V^VO_4^-$, in terms of $H_2V^VO_4^-$ hydrolytic model. Reversing this equation, it is possible to define the formation of $H_2V^VO_4^-$ from $V^VO_2^+$ (Equation S4). This last equation can be exploited to derive the $V^VO_2^+$ hydrolytic model.

$$\log\beta_{1,2}^{H_2V^VO_4^-} \qquad H_2V^VO_4^- + 2H^+ \rightleftarrows V^VO_2^+ + 2H_2O \tag{S3}$$

$$\log\beta_{1,-2}^{V^VO_2^+} = -\log\beta_{1,2}^{H_2V^VO_4^-} \qquad V^VO_2^+ + 2H_2O \rightleftarrows H_2V^VO_4^- + 2H^+ \tag{S4}$$

Combining the Equation S4 with the general Equation S1 for different *p* and *r*, it is possible to define the vanadium(V) hydrolytic species as $V^VO_2^+$ hydrolysis, starting from the $H_2V^VO_4^-$ hydrolytic model. More specifically Equation S4 must be added to Equation S1 as many times as the value of *p* index, representing the nuclearity of the vanadium(V) species formed.

Therefore, to calculate $\log\beta_{p,r}^{V^VO_2^+}$ from $\log\beta_{p,r}^{H_2V^VO_4^-}$ must subtract $\log\beta_{1,2}^{H_2V^VO_4^-}$ of Equation S4 (that account for the formation of $H_2V^VO_4^-$ from $V^VO_2^+$) as many time as the vanadium nuclearity of the considered species (*p* index).

$$\log\beta_{p,r}^{V^VO_2^+} = \log\beta_{p,r}^{H_2V^VO_4^-} - p(\log\beta_{1,2}^{H_2V^VO_4^-}) \tag{S5}$$

Hydrolytic equilibria expressed with the $V^VO_2^+$ formalism and the corresponding formation constants are reported in Table S3. The hydrolytic constants $\log\beta_{p,r}^{V^VO_2^+}$ are calculated from $\log\beta_{p,r}^{H_2V^VO_4^-}$ values, at $I = 0.20$ mol·dm^{-3} and $T = 298.2$ K, reported in Table S2.

Table S2. Dihydrogenvanadanate(V) ion ($H_2V^VO_4^-$) hydrolytic constants ($\log\beta_{p,r}^{H_2V^VO_4^-}$).

$pH_2V^VO_4^- + rH^+ \rightleftarrows (H_2V^VO_4^-)_p(H^+)_r$		
p,r	Species	$\log\beta_{p,r}^{H_2VO_4^-}$ [a]
1,-1	$HV^VO_4^{2-}$	-8.12
1,2	$V^VO_2^+$	6.98
2,-2	$V^V_2O_7^{4-}$	-16.01
2,-1	$HV^V_2O_7^{3-}$	-5.76
2,0	$H_2V^V_2O_7^{2-}$	2.67
4,-2	$V^V_4O_{13}^{6-}$	-9.59
4,-1	$V^V_4O_{13}^{5-}$	-0.38
4,0	$V^V_4O_{12}^{4-}$	9.38
5,0	$V^V_5O_{15}^{5-}$	11.40
10,4	$V^V_{10}O_{28}^{6-}$	50.53
10,5	$HV^V_{10}O_{28}^{5-}$	57.01
10,6	$H_2V^V_{10}O_{28}^{4-}$	61.07
10,7	$H_3V^V_{10}O_{28}^{3-}$	62.84

[a] log β at $I = 0.20$ mol·dm^{-3} and $T = 298.2$ K adapted from ref. [3]

Table S3. Dioxidovanadium(V) ($V^VO_2^+$) hydrolytic constants $\log\beta_{p,r}^{V^VO_2^+}$ calculated from $\log\beta_{p,r}^{H_2V^VO_4^-}$ reported in Table S2 (for $I = 0.20$ mol·dm^{-3} and $T = 298.2$ K).

$pV^VO_2^+ + rH^+ \rightleftarrows (V^VO_2^+)_p(H^+)_r$		
p,r	species	$\log\beta_{p,r}^{V^VO_2^+}$
1,-2	$[V^VO_2(OH)_2]^-$	-7.00
1,-3	$[V^VO_2(OH)_3]^{2-}$	-15.10
2,-6	$[(V^VO_2)_2(OH)_6]^{4-}$	-29.97
2,-5	$[(V^VO_2)_2(OH)_5]^{3-}$	-19.72
2,-4	$[(V^VO_2)_2(OH)_4]^{2-}$	-11.29
4,-10	$[(V^VO_2)_4(OH)_{10}]^{6-}$	-37.51
4,-9	$[(V^VO_2)_4(OH)_9]^{6-}$	-28.30
4,-8	$[(V^VO_2)_4(OH)_8]^{4-}$	-18.54
5,-10	$[(V^VO_2)_5(OH)_{10}]^{5-}$	-23.50
10,-16	$[(V^VO_2)_{10}(OH)_{16}]^{6-}$	-19.27
10,-15	$[(V^VO_2)_{10}(OH)_{15}]^{5-}$	-12.79
10,-14	$[(V^VO_2)_{10}(OH)_{14}]^{4-}$	-8.73
10,-13	$[(V^VO_2)_{10}(OH)_{13}]^{3-}$	-6.96

1.3 8-hydroxyquinolines stability constants

Table S4. 8-HQA protonation constants (log β).

**q8-hqa^{2-} + rH^{+} ⇄ (8-hqa^{2-})$_q$(H^{+})$_r$**			
p,r	species	logβ^{a}	logβ^{b}
1,1	(8-hqa)H^{-}	9.56 ± 0.01	9.55 ± 0.02
1,2	(8-hqa)H_2	13.52 ± 0.01	13.48 ± 0.03

[a] log β from literature at I = 0.20 mol·dm^{-3} and T = 298.2 K [14]

[b] log β experimentally obtained in this work for I = 0.20 mol·dm^{-3} and T = 298.2 K.

2. UV-vis

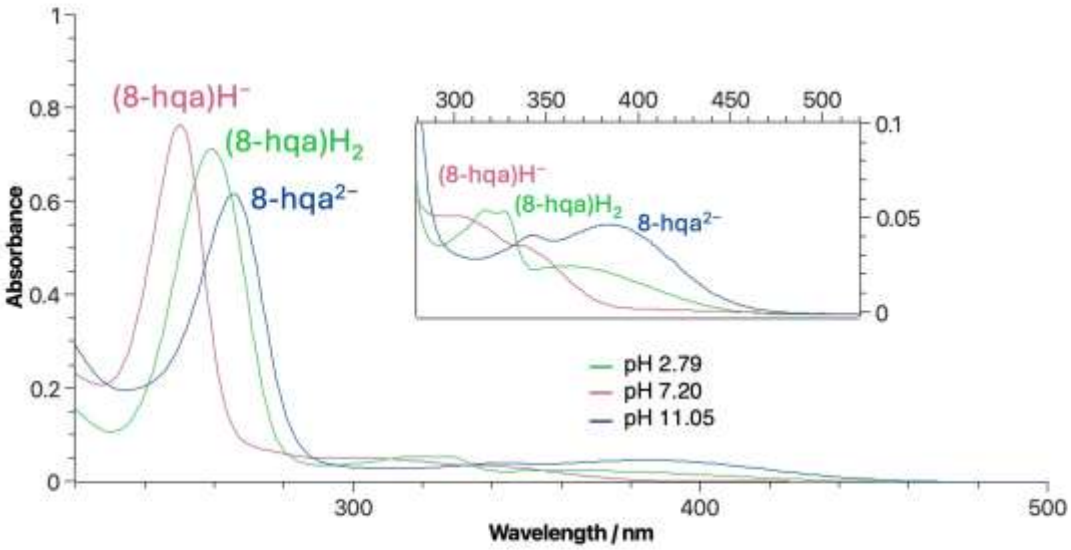

Figure S1. 8-HQA UV-vis spectra – Experimental UV-vis spectra for the three protonation forms of the 8-HQA ($c_{8-\mathrm{HQA}}$ = 0.02 mmol·dm^{-3}, I = 0.2 mol·dm^{-3} in KCl$_{(aq)}$, T = 298.2 K).

3. Potentiometry

Table S5. Concentrations (in mmol·dm^{-3}) of $V^{IV}O^{2+}$, $V^{V}O_2^{+}$ and 8-HQA in the solutions on which the potentiometric titrations were performed (I = 0.2 mol·dm^{-3} in $KCl_{(aq)}$, T = 298.2 K). Titrant: $KOH_{(aq)}$, c_{OH^-}= 0.1 or 0.2 mol·dm^{-3}. Total volume 25 or 50 cm^{-3}.

$c_{V^{IV}O^{2+}}$	c_{8-HQA}	**ratio**	$c_{V^{V}O_2^{+}}$	c_{8-HQA}	**ratio**
M	L	M:L	M	L	M:L
0.2	0.8	1:4	0.2	0.8	1:4
0.2	0.8	1:4	0.3	0.5	1:2
0.2	0.7	1:3	0.3	0.9	1:3
0.2	0.7	1:3	0.4	0.9	1:2
0.2	0.9	1:4	0.4	0.9	1:2
0.2	1.0	1:5	0.4	0.9	1:2
0.3	0.8	1:3	0.4	0.4	1:1
0.3	1.0	1:3	0.4	0.6	1:1
0.3	0.8	1:3	0.4	0.5	1:1
0.3	0.6	1:2	0.4	0.9	1:2
0.3	0.9	1:3	0.5	1.0	1:2
0.3	0.8	1:3	0.5	1.0	1:2
0.3	0.6	1:2	0.5	0.6	1:1
0.3	1.0	1:3	0.8	0.8	1:1
0.4	0.9	1:2	0.8	1.0	1:1
0.4	0.9	1:2			
0.5	0.6	1:1			
0.5	1.0	1:2			
0.6	1.2	1:2			
0.6	0.8	1:1			
0.7	0.8	1:1			
0.7	1.4	1:2			
0.5	1.0	1:2			
0.5	1.0	1:2			
0.5	1.0	1:2			
0.8	1.0	1:1			
0.8	1.0	1:1			
0.8	1.0	1:1			
0.3	1.0	1:3			
0.3	1.0	1:3			
0.3	1.0	1:3			

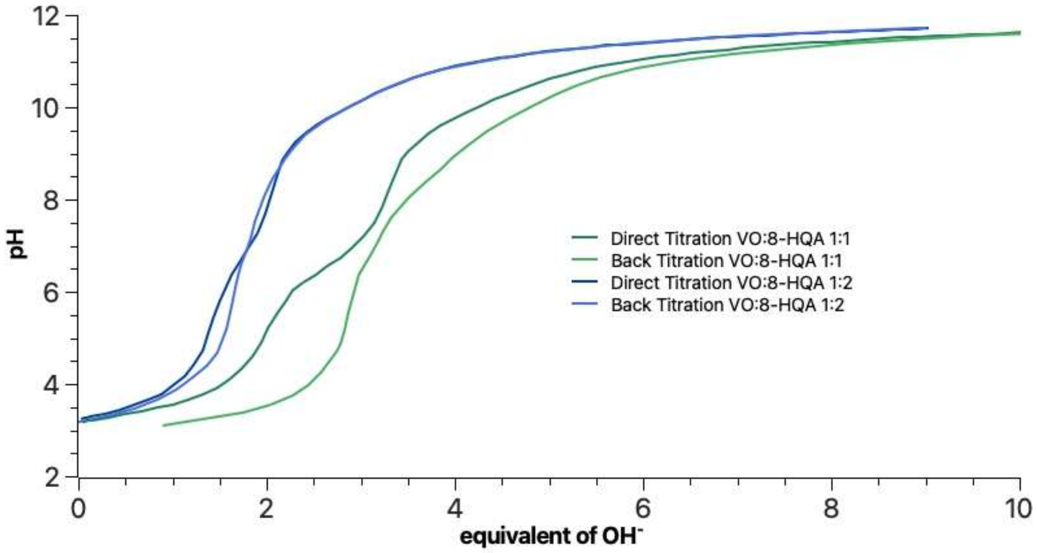

Figure S2. Examples of direct and back-titrations for $V^{IV}O^{2+}$/8-HQA aqueous solutions ($c_{V^{IV}O^{2+}} = c_{8-HQA} =$ 1.0 mmol·dm^{-3} (green) $c_{V^{IV}O^{2+}}$ = 0.5 mmol·dm^{-3}, c_{8-HQA} = 1.0 mmol·dm^{-3} (blue)) at I = 0.2 mol·dm^{-3} in $KCl_{(aq)}$ and T = 298.2 K.

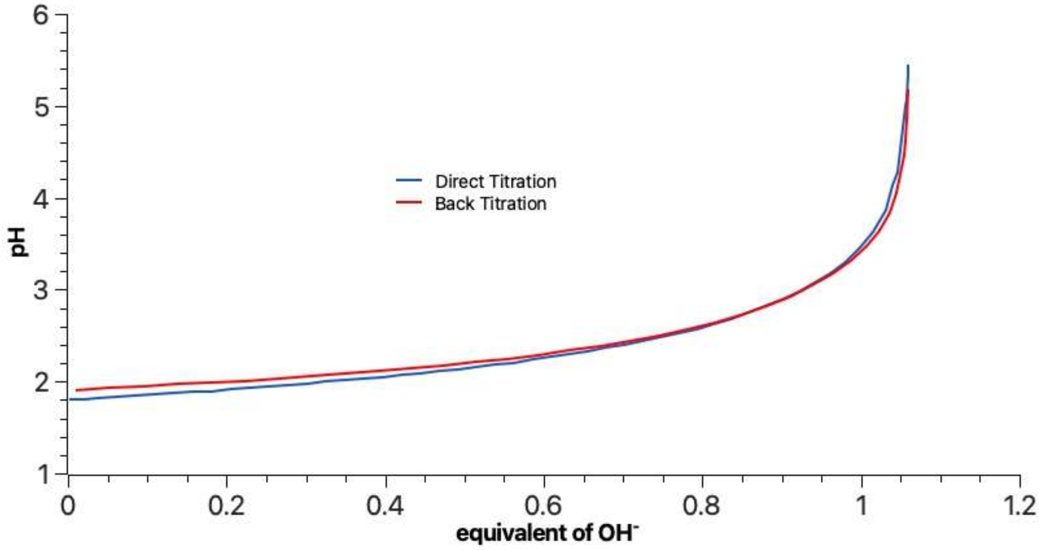

Figure S3. Direct and back-titration curves, respectively in blue and red, for $V^{IV}O^{2+}$/8-HQA aqueous solution ($c_{V^{IV}O^{2+}}$ = 0.5 mmol·dm^{-3}, c_{8-HQA} = 1.0 mmol·dm^{-3}, I = 0.2 mol·dm^{-3} in $KCl_{(aq)}$, T = 298.2 K). The direct titration was limited to pH ~ 6, and the back-titration started immediately after.

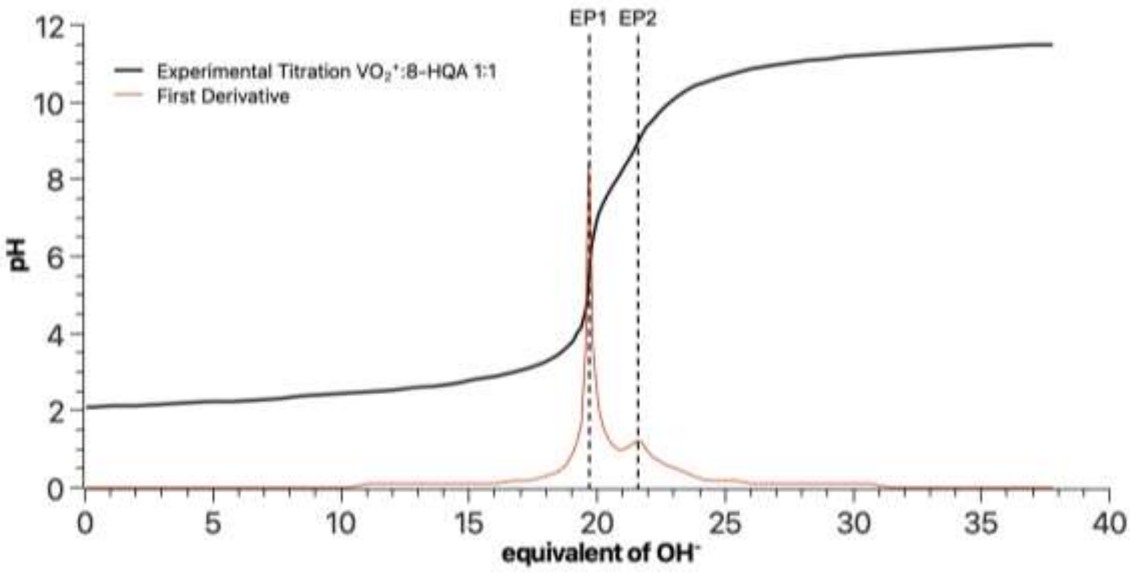

Figure S4. Experimental direct titration curve (black) and first derivative (red) for $V^VO_2^+$/8-HQA aqueous solution ($c_{V^VO_2^+}$ = 0.41 mmol·dm^{-3}, c_{8-HQA} = 0.44 mmol·dm^{-3}, c_{HCl} = 8.8 mmol·dm^{-3}, I = 0.2 mol·dm^{-3} in $KCl_{(aq)}$, T = 298.2 K).

4. ESR

Table S6. Calculated hyperfine coupling constant values ($A_{\parallel}^{calc}$) and schematic representations of possible isomers of oxidovanadium(IV) complexes of 8-HQA. $A_{\parallel}^{calc}$ were predicted by the additivity relationship [15] considering the supposed donor set.

**$V^{IV}O$(8-hqa)**					
**Species**		**Equatorial donor set**		**$A_{\parallel}^{calc}$**	**Structure**
		Donor	ν ($\times 10^{-4}$ cm^{-1})	($\times 10^{-4}$ cm^{-1})	
a	[$V^{IV}O$(8-hqa)$(H_2O)_3$]	H_2O	45.7	170.3	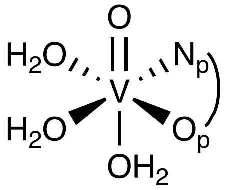
		H_2O	45.7		
		N_{pyr}	40.3		
		O_{phen}	38.6		
b	[$V^{IV}O$(8-hqa)$(H_2O)_2$]	H_2O	45.7	167.3	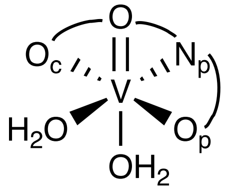
		O_{carb}	42.7		
		N_{pyr}	40.3		
		O_{phen}	38.6		
**[$V^{IV}O$(8-hqa)(OH)]$^{-}$**					
**Species**		**Equatorial donor set**		**$A_{\parallel}^{calc}$**	**Structure**
		Donor	ν ($\times 10^{-4}$ cm^{-1})	($\times 10^{-4}$ cm^{-1})	
c	[$V^{IV}O$(8-hqa)(OH)$(H_2O)_2$]$^{-}$	H_2O	45.7	163.3	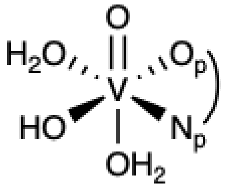
		N_{pyr}	40.3		
		O_{phen}	38.6		
		OH	38.7		
d	[$V^{IV}O$(8-hqa)(OH)(H_2O)]$^{-}$	O_{carb}	42.7	160.3	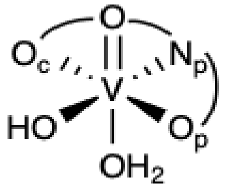
		N_{pyr}	40.3		
		O_{phen}	38.6		
		OH	38.7		

5. DFT calculations

5.1 $[V^{IV}O(H_2O)_5]^{2+}$ structure

To evaluate the quality and accuracy of our DFT approach, the $[V^{IV}O(H_2O)_5]^{2+}$ ion was characterized. Extensive literature on this system provided several benchmarks.[16–19] Figure S6 presents the optimized structure of $[V^{IV}O(H_2O)_5]^{2+}$ computed at the ωB97X-3c level with the implicit solvation model (refer to the DFT Calculations section in the Experimental Section 2.8 for details). Table S7 summarizes the geometrical and ESR parameters obtained in this study alongside those reported in previous works ESR. The calculated V=O distance is in perfect agreement with the previous work from Lagostina *et al.* [18], and in a good agreement with the experimental X-ray diffraction measurements.[16,20] Similarly, distances between the Vanadium and the water molecules in the axial and equatorial positions nicely match both previous theoretical and experimental results, with just a small average overestimation of 0.01 Å. Both $A_\perp$ and $A_\parallel$ are correctly described by the computational procedure, with a slight improvement on the calculation of $A_\perp$ with respect to the one presented by Lagostina *et al.* [18] On the other hand, the prediction of *g*-tensors at DFT level is a non-trivial task. For the sake of simplicity, the *g*-tensors at DFT level was computed, although Lagostina *et al.* [18] showed than an improvement of estimated *g*-tensors can be achieved by a CASSCF[CAS(5,1)]/NEVPT2 treatment. Nevertheless, previous studies have demonstrated good agreement between experimental and DFT-computed *g*-tensors for oxidovanadium(IV) complexes.[21–23] The computed values of $g_\perp$ and $g_\parallel$ are generally slightly underestimated compared to experimental data, with deviations ranging from 0.006 to 0.023. However, the computed $g_\parallel$ matches perfectly with the value reported by Lagostina *et al.*[18] Although DFT methods are not always optimal for predicting magnetic properties, the present calculations of both *g*-tensor and hyperfine coupling constants confirm that the adopted computational procedure provides reliable predictions of ESR parameters for oxidovanadium(IV) complexes.

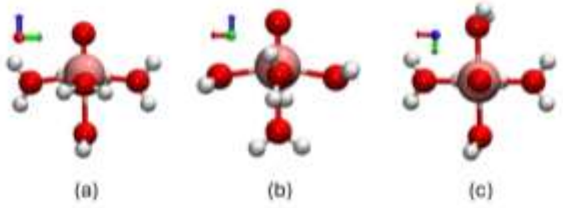

Figure S5. $[VO(H_2O)_5]^{2+}$ ion calculated structure – Different view of the $[V^{IV}O(H_2O)_5]^{2+}$ ion optimized at ωB97X-3c level. **(a)** Equatorial view along the x-axis. **(b)** Equatorial view along the y-axis. **(c)** Axial view along the z-axis. Color Legend: Pink - Vanadium; Red - Oxygen; White - Hydrogen.

Table S7. $[V^{IV}O(H_2O)_5]^{2+}$ ion geometrical and ESR parameters calculated and experimentally determined in this paper and in previous works.[16,18–20,23]

$[V^{IV}O(H_2O)_5]^{2+}$		$V=O$	$V\text{-}O_{H_2O}$	$g_\perp$	$g_\parallel$	$A_\perp$ ($\times10^{-4}$ cm^{-1})	$A_\parallel$ ($\times10^{-4}$ cm^{-1})	Ref.
This work	Computed	1.565	2.046–2.053[a]; 2.226[b]	1.968	1.91	69	184	-
	Experimental	-	-	1.974	1.928	69	182	-
Previous works	Computed	1.565<br>1.561	2.052-2.056[a]; 2.212[b]<br>2.098[a]; 2.330[b]	1.983<br>1.902	1.91<br>1.804	67<br>58	184<br>19	18<br>23
	Experimental	1.569<br>1.577	2.023-2.030[a]; 2.183[b]<br>2.026-2.027[a]; 2.175[b]	1.979	1.934	69	182	16,18–20

[a] Distances for equatorial water molecules
[b] Distance for axial water molecule

5.2 [V^{IV}O(8-hqa)] structure

To shed some light on the [V^{IV}O(8-hqa)] complex a multi-step computational procedure was adopted. Firstly, the geometry was optimized at HF-3c to create the initial minima from which the conformational exploration starts. In order to elucidate the geometry of the complex, six water molecules were incorporated to simulate the primary coordination sphere of the metal center and assess the influence of hydration on complex formation. Moreover, to evaluate preference between a bidentate or tridentate behavior of 8-HQA, two distinct initial configurations were constructed: one in which the COO^{-} group in directly involved in coordination (*tridentate* configuration) and another in which a water molecule replaces the COO^{-} group in the coordination sphere (*bidentate* configuration). In Figure S7a,b are reported structures optimized at HF-3c level. From these two geometries, the conformational space was explored using the GOAT algorithm. The *tridentate* configuration resulted in only 3 conformers in the range of 25 kJ/mol. On the contrary, the *bidentate* configuration shown a higher conformational freedom, with 15 conformers in the same energy ensemble. In Figure S7c,d are reported the global minima found at HF-3c for *tridentate* configuration (c) and *bidentate* configuration (d). In Table S8 are reported the geometrical and energetical data of the structures reported in Figure S7. As shown in Table S8, the V-O(COO^{-}/H_2O) distance are identical for both geometries, while the V=O and V-N distance are 0.1 and 0.2 Å longer for the *bidentate* configuration, respectively. The major difference observed is for the phenolic oxygen, where in the case of the *tridentate* configuration complex (see Figure S7c) the distance is 0.3 Å larger than the *bidentate* configuration (see Figure S7d). Energy calculations at the HF-3c level reveal that the *bidentate* configuration is more stable than the *tridentate* configuration one by 27.5 kJ/mol. Although HF-3c is a cost-effective method that reliably generates initial geometries, its energy estimates are notably sensitive to the small basis sets inherent to the -3c approximation.[24] Nevertheless, given the extensive number of optimizations required for a comprehensive conformational search, HF-3c was the most practical approach for obtaining plausible geometries at a minimal computational expense. To improve the reliability of these results, we computed a single point energy calculation using the ωB97X-3c on the HF-3c geometry (*i.e.* ωB97X-3c // HF-3c). The former, is a new composite range-separated hybrid functional which demonstrated to be effective also for transition metal complexes.[25] Table S8 illustrates that the energy trend computed with the ωB97X-3c method is completely reversed relative to the HF-3c results, with the *tridentate* configuration exhibiting an approximate 90 kJ/mol stabilization compared to its *bidentate* configuration. These conflicting trends preclude a definitive conclusion regarding the relative stabilities of the two configurations. Consequently, both global minima were reoptimized using the more accurate ωB97X-3c method. The new geometries are illustrated in Figure S7e,f, while their corresponding geometrical and energetical features are in Table

S8. In general, for both structures, the V=O and V-N distances are shorter, while the V-O(phenol) and V-O(COO^{-}/H_2O) are stretched with respect to the corresponding HF-3c structure. The new computed energy difference highlights a predominant stability of the *bidentate* configuration, with a difference of 150 kJ/mol. Interestingly, in the *tridentate* configuration, both V=O bond and the V–O(phenol) bond, are respectively 0.11 Å and 0.5 Å longer compared to the *bidentate* configuration. These structural differences could explain the large energy gap between the two. Although the initial geometries were similar (see Figure S7c,d), the optimized structures differ significantly (see Figure S7e,f). This indicates that the potential energy surface described by ωB97X-3c is very different from that of HF-3c, meaning that a global minimum found with HF-3c may not be the global minimum when reoptimized using ωB97X-3c. To overcome this problem, all the final ensembles found for both structures were reoptimized at *ω*B97X-3c, considering in total 18 geometries. The new global minima are reported in Figure S7g,h. In agreement with the global minima found at HF-3c level, the V=O distance is almost identical between the two structures, while the V-N distance is slightly larger for the *bidentate* configuration. Surprisingly, both V-O(phenol) and V-O(COO^{-}/H_2O) distances are very similar between the two complexes. The final energy difference between the two structures is 0.96 kJ/mol at *ω*B97X-3c. Finally, to assess the quality of this result a single point calculation using the highly accurate DLPNO-B2PLYP method [26] coupled with a def2-TZVPP basis set was performed. As shown in Table S8, the energy difference between the structures is very similar to the one obtained at *ω*B97X-3c level, with *bidentate* configuration being only 3.3 kJ/mol more stable than the *tridentate* one.

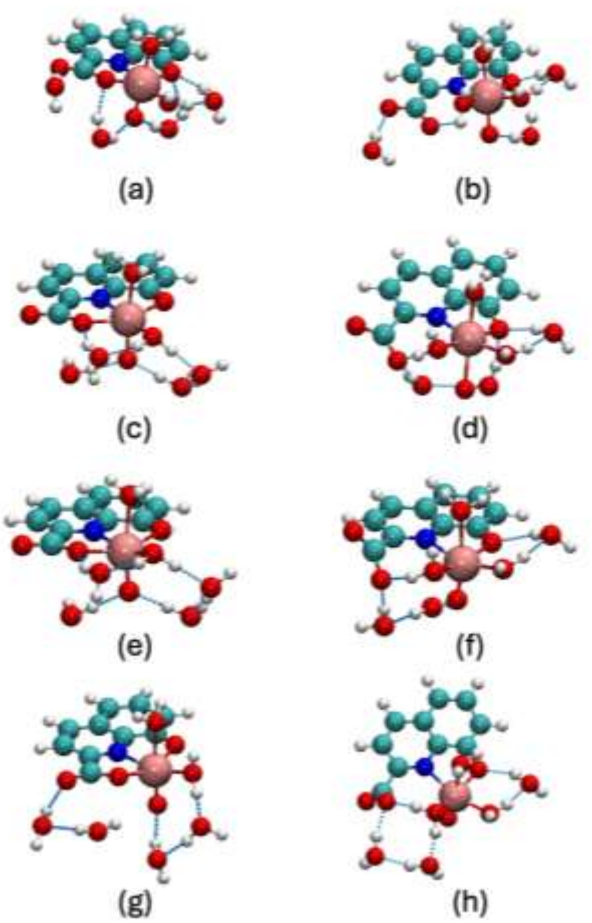

Figure S6. Calculated structures of [V^{IV}O(8-hqa) $(H_2O)_n$] × $(H_2O)_{6-n}$ – (left column) The [V^{IV}O(8-hqa) $(H_2O)_2$] × $(H_2O)_4$ complex in the *tridentate* configuration. **(right column)** The [V^{IV}O(8-hqa) $(H_2O)_3$] × $(H_2O)_3$ complex in the *bidentate* configuration. **(a-b)** Guessed structures used as starting point to explore the conformational space. Geometries were optimized at HF-3c level: both structures are minima of the PES. **(c-d)** Global minima obtained at HF-3c level. **(e-f)** Re-optimization of the c-d global minima at ωB97X-3c. **(g-h)** New Global minima obtained after the new ranking at ωB97X-3c level. Dashed blue lines represent hydrogen bonds. Color legend: pink - vanadium; red - oxygen; blue - nitrogen; cyan - carbon; white - hydrogen.

Table S8. Distances and energy difference referring to the structures reported in Figure S7. Column V-O(COO^-/H_2O) refers to the same ligand position in the case of *tridentate* or *bidentate* configuration. The unit reported in kJ/mol is the absolute energy difference between the two structures.

Structure	Method	V=O	V-N	V-O (phenol)	V-O (COO^-/H_2O)	N. H-bonds
**a (tri)**	HF-3c[a]	1.8	2.02	1.93	1.97	7
**b (bi)**	HF-3c[a]	1.93	2.20	2.04	2.15	5
**c (tri)**	HF-3c[a]	1.98	2.10	2.37	2.06	5
**d (bi)**	HF-3c[a]	2.18	2.18	2.02	2.08	6
**c − d**	HF-3c[a]	27.5 kJ/mol				
**c − d**	***ω***B97X-3c // HF-3c	-87.7 kJ/mol				
**e (tri)**	***ω***B97X-3c[a]	1.679	2.083	2.437	2.086	6
**f (bi)**	***ω***B97X-3c[a]	1.562	2.151	1.922	2.09	5
**e − f**	***ω***B97X-3c[a]	150 kJ/mol				
**g (tri)**	***ω***B97X-3c[a]	1.58	2.0	1.983	2.05	5
**h (bi)**	***ω***B97X-3c[a]	1.565	2.143	1.933	2.04	6
**g − h**	***ω***B97X-3c[a]	0.96 kJ/mol				
**g − h**	DLPNO-B2PLYP[c] // ***ω***B97X-3c	3.3 kJ/mol				

Distances are reported in Å

[a] Geometry optimized with this method.

[b] Single point calculation.

[c] def2-TZVPP basis set was coupled with the DLPNO-B2PLYP method.

5.3 8-HQA UV-vis spectra calculation

Geometries were optimized at ωB97X-3c level, adopting the CPCM solvation model. The UV/Vis simulation were obtained at ωB97X level coupled with a def2-TZVP basis set and are reported in Figure S8. In general, all the spectra are well described by the computed excited wavelengths. The two relevant peaks around ~ 260 and ~ 200 nm (λ ~ 60 and ~ 0 nm in Relative Wavelength scale) are associated to the HOMO-LUMO transition for all the different protonation form of 8-HQA reported. In Figure S9 are reported the Natural Transition Orbitals (NTO) associated to the two transitions for the fully deprotonated form 8-hqa^{2-}. As shown, the electron mainly occupies a π-orbital delocalizing all over the conjugated system for both the HOMO and LUMO orbital, describing as expected a $\pi - \pi^*$transition.

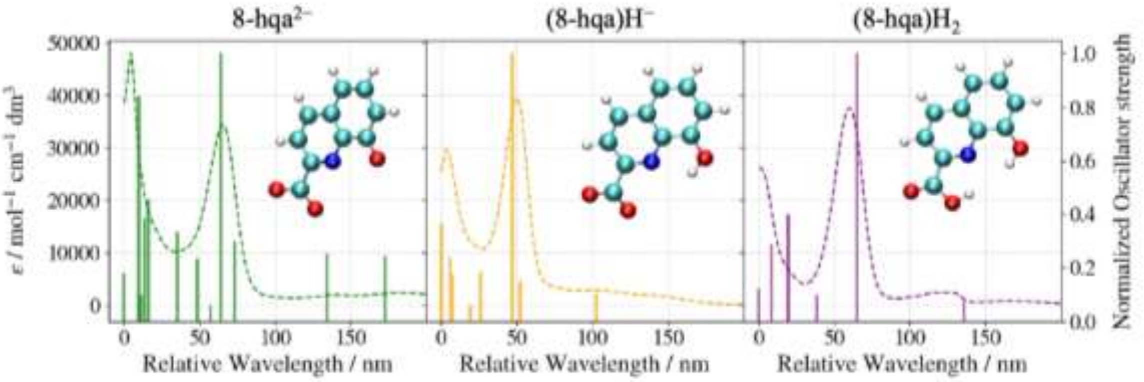

Figure S7. Computed 8-HQA UV-vis spectrum – Comparison between experimental and computed UV-vis spectra for the three different protonated states 8-HQA: **(left)** fully deprotonated 8-hqa^{2-}, **(center)** mono-protonated (8-hqa)H^{-} and **(right)** doubly-protonated state (8-hqa)H_2^{-}. Experimental results are shown with dashed lines, while the computed transitions are reported with continuous lines. Relative positions of the peaks were calculated using the first peak of the computational data and experimental data as reference points. The resulting spectra are plotted on a relative wavelength scale, maintaining the original intensities. Only normalized computational intensities with a value above 0.05 were included in the plot.

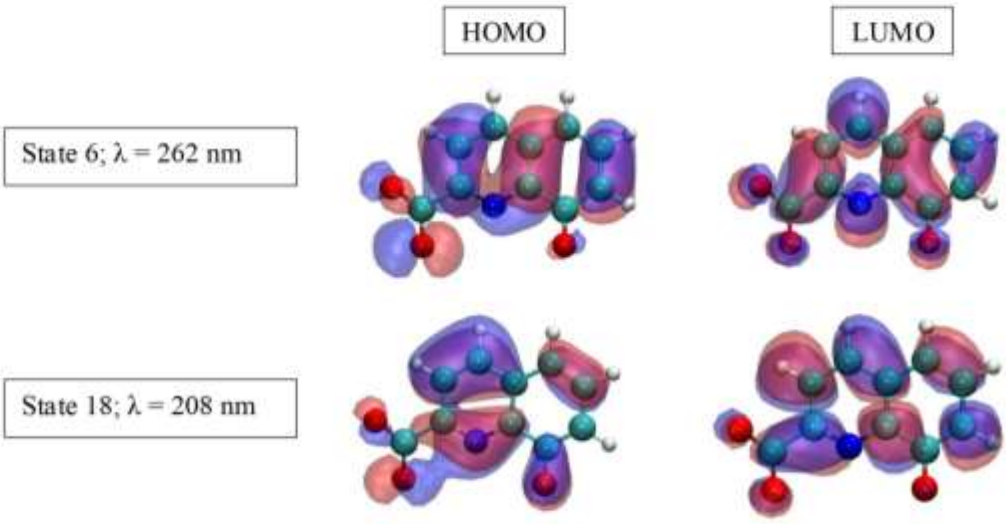

Figure S8. Fully deprotonated 8-HQA NTO – Representation of HOMO and LUMO fully deprotonated 8-hqa^{2-} NTO for the two most intense peaks reported in Figure S8, left panel.

5.4 $V^{IV}O^{2+}$/8-HQA UV-vis spectra calculation

Figure S9 shows the simulated and experimental UV-vis spectra of $[V^{IV}O(8\text{-hqa})(H_2O)_n]\times(H_2O)_{6-n}$ for both previously reported global minima (see Figure S7g,h). Relative positions of the peaks were calculated using the first peak of the computational data and experimental data as reference points. The simulated transitions that best reproduce the experimental UV-vis spectra correspond to those for the *bidentate* configuration. Nevertheless, transitions associated with the *tridentate* configuration, such as those at $\lambda \sim 310$ nm ($\lambda \sim 110$ nm in Relative Wavelength scale) contribute to the experimental spectra. No definitive preference for a single configuration can be inferred from the UV-vis spectra simulation. For details on the molecular orbitals involved in the main transitions, one may refer to Figures S10 and S11.

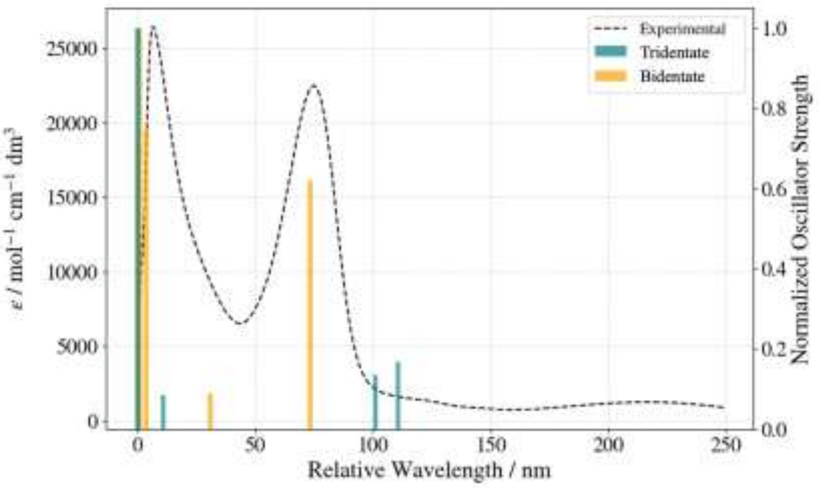

Figure S9. Comparison between experimental and computed UV-vis spectra for the two feasible $[V^{IV}O(8\text{-hqa})(H_2O)_n] \times (H_2O)_{6-n}$ structures. Experimental results are shown with dashed line, while the computed transitions are reported with continuous lines. Relative positions of the peaks were calculated using the first peak of the computational data and experimental data as reference points. The resulting spectra are plotted on a relative wavelength scale, maintaining the original intensities. For the sake of clarity, only normalized computational intensities with a value above 0.05 were included in the plot.

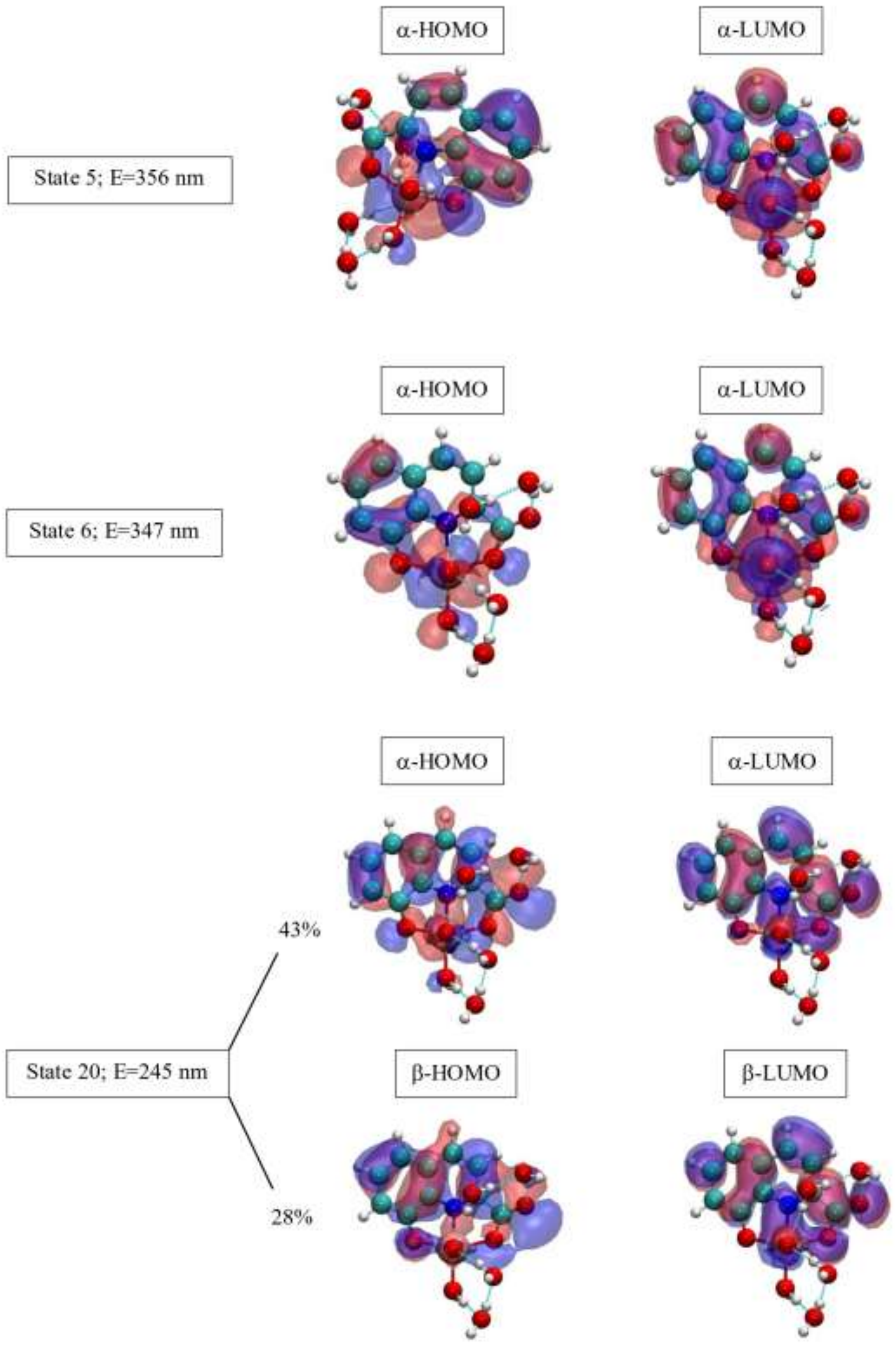

Figure S10. COO$^-$-binding oxidovanadium (IV) complex NTO – Representation of HOMO and LUMO NTO for the *tridentate* configuration of $V^{IV}O$(8-hqa). The percentage value for each HOMO-LUMO pair describes the weight of the transition in the NTO. Only the most important couples were showed.

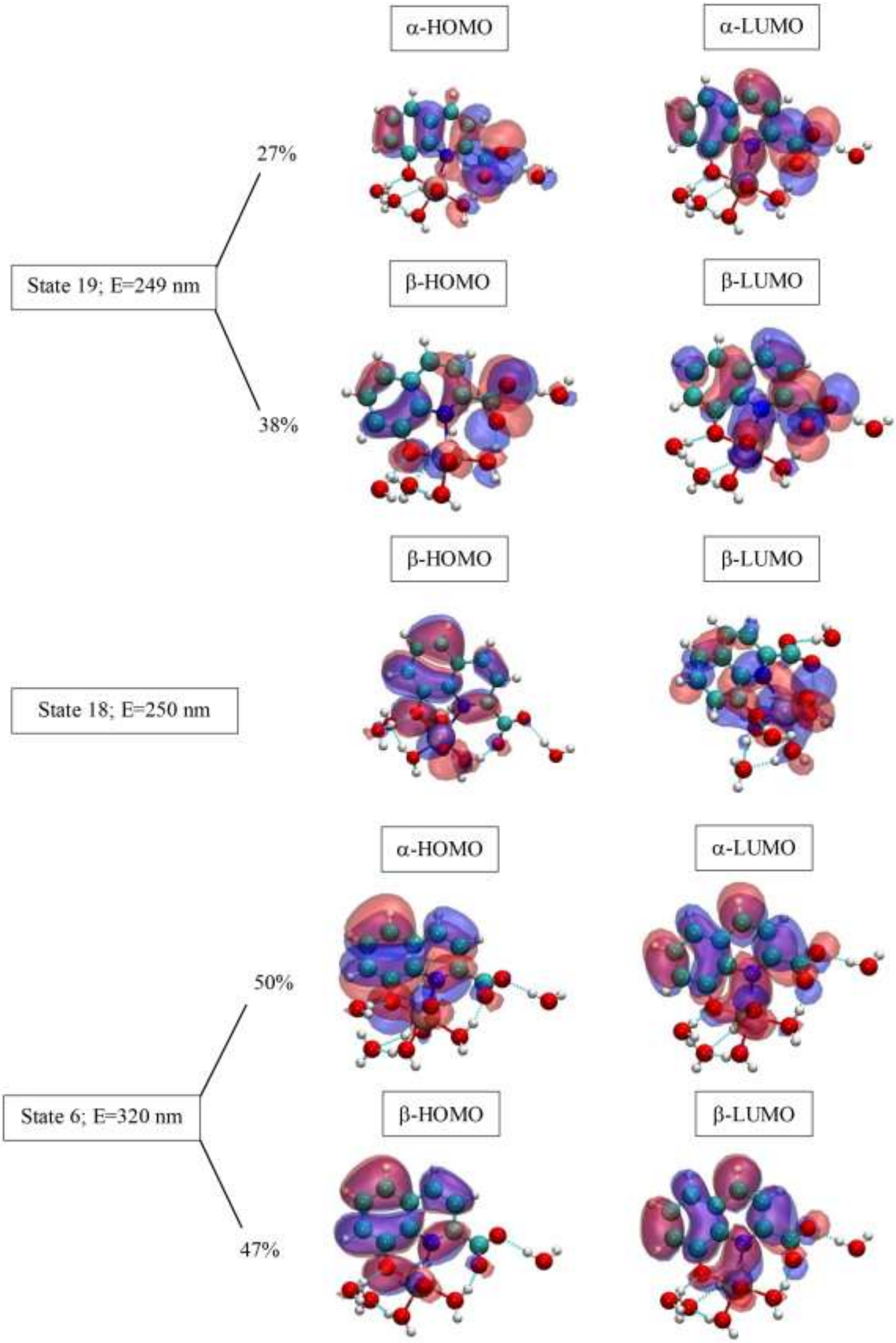

Figure S11. H_2O-binding oxidovanadium(IV) complex NTO – Representation of HOMO and LUMO NTO for the *bidentate* configuration of $V^{IV}O$(8-hqa). The percentage value for each HOMO-LUMO pair describes the weight of the transition in the NTO. Only the most important couples were showed.

5.5 $V^{IV}O^{2+}$/8-HQA ESR spectra calculation

Concerning the ESR measurements, in Table S9 is reported the Spin-Hamiltonian parameters computed for the *tridentate* and *bidentate* configurations, compared with the experimental ones. With a very similar computed $g_{\perp}$, both species align with the experimental.; Nevertheless, $g_{\parallel}$ is best reproduced by the *bidentate* structure. Less clear is the analysis of the *A*-tensors. For both structures the discrepancies between computed and experimental values of the hyperfine coupling constants $A_{\perp}$ and $A_{\parallel}$ do not allow to discriminate between *tridentate* and *bidentate* configurations.

Table S9. Spin-Hamiltonian parameters obtained for the computed $[V^{IV}O(8\text{-hqa})\ (H_2O)_n] \times (H_2O)_{6-n}$ complexes and the experimental reported in this work.

$[V^{IV}O(8\text{-hqa})\ (H_2O)_n] \times (H_2O)_{6-n}$	$g_{\perp}$	$g_{\parallel}$	$A_{\perp}$ ($\times 10^{-4}$ cm^{-1})	$A_{\parallel}$ ($\times 10^{-4}$ cm^{-1})
Tridentate	1.971	1.921	50	164
Bidentate	1.974	1.931	55	160
Experimental	1.974	1.933	60-61	171-172

6. NMR

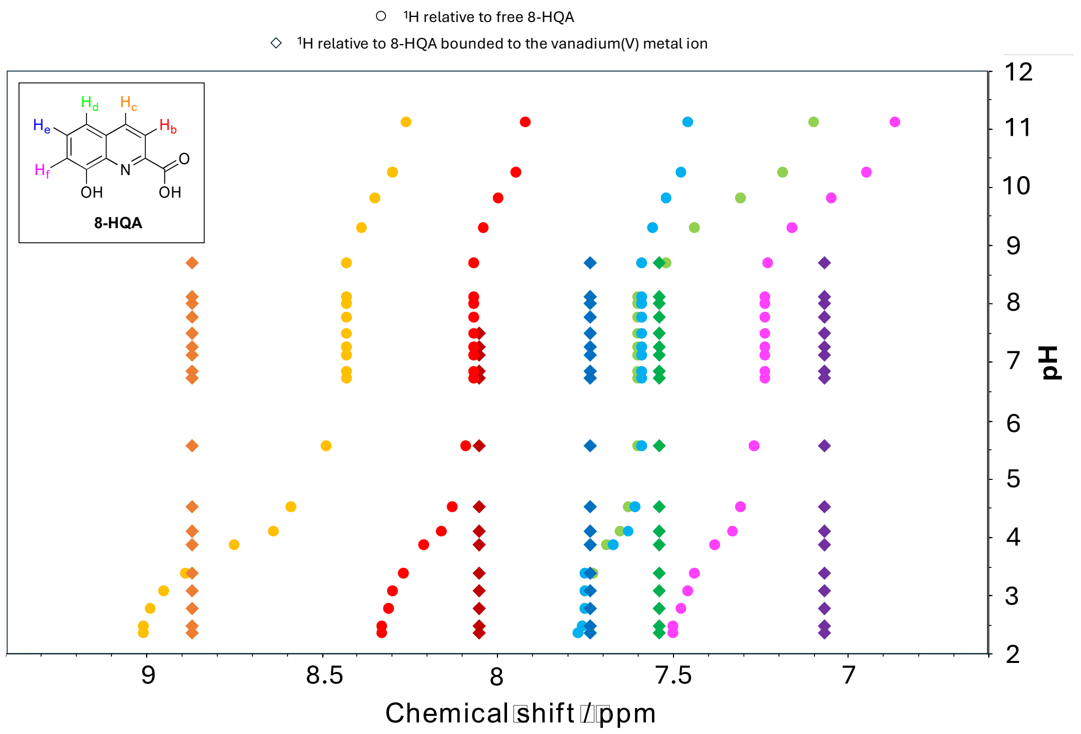

Figure S12. $V^VO_2^+$/8-HQA ^{1}H-NMR spectra – pH dependent ^{1}H-NMR spectra for aqueous solution mixture $V^VO_2^+$:8-HQA 0.5:1 mmol·dm^{-3} (I = 0.2 mol·dm^{-3} in $KCl_{(aq)}$, T = 298.2 K). In the spectra can be observed the ^{1}H-NMR signals for the 8-HQA bound to dioxidovanadium(V) (◆) and the ^{1}H-NMR signal for the free 8-HQA in solution(●). Spectra were recorded 24h after the preparation of the samples.

7. ESI-MS

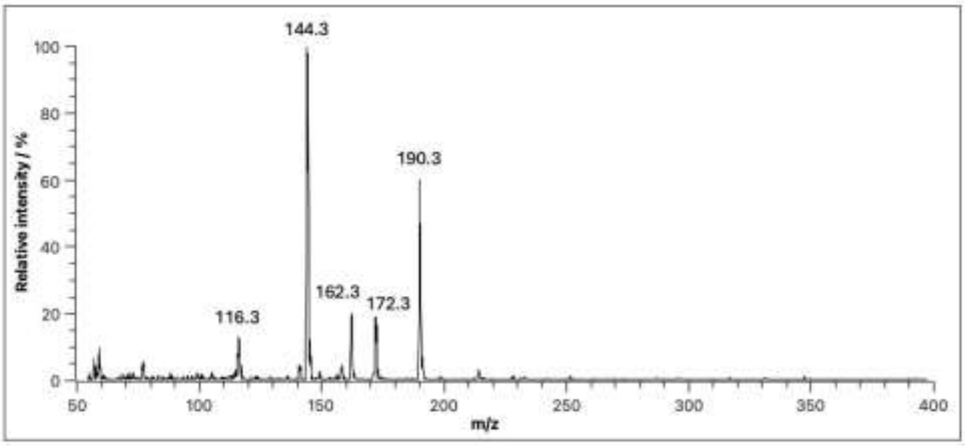

Figure S13. 8-HQA ESI-MS spectra – ESI-MS(+) spectrum of 8-HQA solution ($c_{8-\mathrm{HQA}}$= 0.02 mmol·dm^{-3}) at pH = 5.

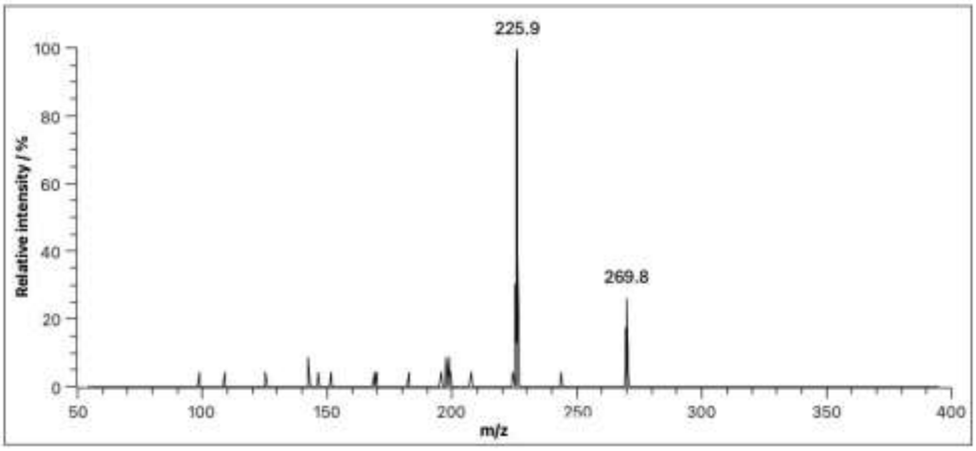

Figure S14. $V^VO_2^+$/8-HQA ESI-MS^2 spectra – ESI-MS^2(−) spectrum of $V^VO_2^+$:8-HQA solution at pH 5 a ($c_{V^VO_2^+}$= $c_{8-\mathrm{HQA}}$= 0.02 mmol·dm^{-3}). Product of *m/z* 269.9.

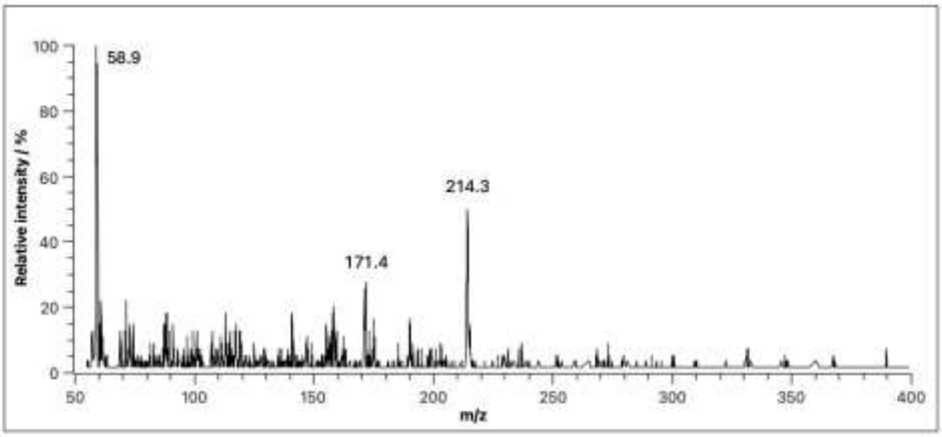

Figure S15. $V^VO_2^+$/8-HQA ESI-MS spectra – ESI-MS(+) spectrum of a $V^VO_2^+$/8-HQA solution at pH = 5 ($c_{V^VO_2^+}$= $c_{8-\mathrm{HQA}}$= 0.02 mmol·dm^{-3}).

References

(1) Henry, R. P.; Mitchell, P. C. H.; Prue, J. E. Hydrolysis of the Oxovanadium(IV) Ion and the Stability of Its Complexes with the 1,2-Dihydroxybenzenato(2–) Ion. *J. Chem. Soc., Dalton Trans.* **1973**, No. 11, 1156–1159. 10.1039/DT9730001156.

(2) Costa Pessoa, J. Thirty Years through Vanadium Chemistry. *J Inorg Biochem* **2015**, *147*, 4–24. 10.1016/j.jinorgbio.2015.03.004.

(3) Elvingson, K.; González Baró, A.; Pettersson, L. Speciation in Vanadium Bioinorganic Systems. 2. An NMR, ESR, and Potentiometric Study of the Aqueous H^+−Vanadate−Maltol System. *Inorg Chem* **1996**, *35* (11), 3388–3393. 10.1021/ic951195s.

(4) Pettersson, L. Speciation in Peroxovanadate Systems. *Coord Chem Rev* **2003**, *237* (1–2), 77–87. 10.1016/S0010-8545(02)00223-0.

(5) Gorzsás, A.; Andersson, I.; Pettersson, L. Speciation in Aqueous Vanadate–Ligand and Peroxovanadate–Ligand Systems. *J Inorg Biochem* **2009**, *103* (4), 517–526. 10.1016/j.jinorgbio.2008.12.006.

(6) Andersson, I.; Gorzsás, A.; Pettersson, L. Speciation in the Aqueous $H^+/H_2VO_4^-/H_2O_2$/Picolinate System Relevant to Diabetes Research. *Dalton Trans.* **2004**, *4* (3), 421–428. 10.1039/B313424E.

(7) Schmidt, H.; Andersson, I.; Rehder, D.; Pettersson, L. A Potentiometric and ^{51}V NMR Study of the Aqueous $H^+/H_2VO_4^-/H_2O_2$/L-α-Alanyl-L-Histidine System. *Chemistry – A European Journal* **2001**, *7* (1), 251–257. 10.1016/S0010−8545(01)00437−4.

(8) Andersson, I.; Angus-Dunne, S.; Howarth, O.; Pettersson, L. Speciation in Vanadium Bioinorganic Systems. *J Inorg Biochem* **2000**, *80* (1–2), 51–58. 10.1016/S0162-0134(00)00039-8.

(9) Rehder, D. The (Biological) Speciation of Vanadate(V) as Revealed by ^{51}V NMR: A Tribute on Lage Pettersson and His Work. *J Inorg Biochem* **2015**, *147*, 25–31. 10.1016/j.jinorgbio.2014.12.014.

(10) Foti, C.; Gianguzza, A.; Sammartano, S. A Comparison of Equations for Fitting Protonation Constants of Carboxylic Acids in Aqueous Tetramethylammonium Chloride at Various Ionic Strengths. *J Solution Chem* **1997**, *26* (6), 631–648. 10.1007/BF02767633.

(11) Daniele, P. G.; De Robertis, A.; De Stefano, C.; Sammartano, S. Ionic Strength Dependence of Formation Constants. XIII. A Critical Examination of Preceding Results. In *Miscellany of Scientific Papers in Honour of Enric Casassas, Professor of Analytical Chemistry, Universitat Autonoma de Barcelona, Bellaterra, Spain*; Alegret, S., Arias, J. J., Barceló, D., Casal, J., Rauret, G., Eds.; 1991; pp 121–126.

(12) Baes, C. F. J.; Mesmer, R. E. *The Hydrolysis of Cations*; Wiley, 1976.

(13) Hydrolysis of Metal Ions; Brown, P. L., Ekberg, C., Eds.; Wiley, 2016. 10.1002/9783527656189.

(14) Gama, S.; Frontauria, M.; Ueberschaar, N.; Brancato, G.; Milea, D.; Sammartano, S.; Plass, W. Thermodynamic Study on 8-Hydroxyquinoline-2-Carboxylic Acid as a Chelating Agent for Iron Found in the Gut of Noctuid Larvae. *New Journal of Chemistry* **2018**, *42* (10), 8062–8073. 10.1039/C7NJ04889K.

(15) Smith, T. S.; LoBrutto, R.; Pecoraro, V. L. Paramagnetic Spectroscopy of Vanadyl Complexes and Its Applications to Biological Systems. *Coord Chem Rev* **2002**, *228* (1), 1–18. 10.1016/S0010-8545(01)00437-4.

(16) Krakowiak, J.; Lundberg, D.; Persson, I. A Coordination Chemistry Study of Hydrated and Solvated Cationic Vanadium Ions in Oxidation States +III, +IV, and +V in Solution and Solid State. *Inorg Chem* **2012**, *51* (18), 9598–9609. 10.1021/ic300202f.

(17) Micera, G.; Garribba, E. Is the Spin-Orbit Coupling Important in the Prediction of the 51V Hyperfine Coupling Constants of $V^{IV}O^{2+}$ Species? ORCA versus Gaussian Performance and Biological Applications. *J Comput Chem* **2011**, *32* (13), 2822–2835. 10.1002/jcc.21862.
(18) Lagostina, V.; Carniato, F.; Esteban-Gómez, D.; Platas-Iglesias, C.; Chiesa, M.; Botta, M. Magnetic and Relaxation Properties of Vanadium(IV) Complexes: An Integrated ^{1}H Relaxometric, EPR and Computational Study. *Inorg Chem Front* **2023**, *10* (7), 1999–2013. 10.1039/d2qi02635j.
(19) Atherton, N. M.; Shackleton, J. F. Proton ENDOR of $VO(H_2O)_5^{2+}$ in $Mg(NH_4)_2(SO_4)_2)26H_2O$. *Mol Phys* **1980**, *39* (6), 1471–1485. https://doi.org/10.1080/00268978000101211.
(20) Magnussen, M.; Brock-Nannestad, T.; Bendix, J. Pentaaquaoxovanadium(IV) Bis(Trifluoromethanesulfonate). *Acta Crystallogr C* **2007**, *63* (2), m51–m53. 10.1107/S010827010605219X.
(21) Bottini, R. C. R.; Fachini, L. G.; Baptistella, G. B.; Stinghen, D.; Santana, F. S.; Briganti, M.; Ribeiro, R. R.; Soares, J. F.; Sá, E. L.; Nunes, G. G. An Unsymmetrical Mixed-Valence Oxidovanadium(IV/V) Binuclear Complex: Synthesis, Characterization, DFT Studies, and Bromoperoxidase Activity. *Inorganica Chim Acta* **2022**, *537*, 120947. 10.1016/j.ica.2022.120947.
(22) Maurelli, S.; Berlier, G.; Chiesa, M.; Musso, F.; Corà, F. Structure of the Catalytic Active Sites in Vanadium-Doped Aluminophosphate Microporous Materials. New Evidence from Spin Density Studies. *The Journal of Physical Chemistry C* **2014**, *118* (34), 19879–19888. 10.1021/jp505896x.
(23) Costa Pessoa, J.; Calhorda, M. J.; Cavaco, I.; Correia, I.; Duarte, M. T.; Felix, V.; Henriques, R. T.; Piedade, M. F. M.; Tomaz, I. Molecular Modelling Studies of N-Salicylideneamino Acidato Complexes of Oxovanadium(IV). Molecular and Crystal Structure of a New Dinuclear LOV^{IV}–O–$V^{V}OL$ Mixed Valence Complex. *Journal of the Chemical Society, Dalton Transactions* **2002**, No. 23, 4407. 10.1039/b205843j.
(24) Sure, R.; Grimme, S. Corrected Small Basis Set Hartree-Fock Method for Large Systems. *J Comput Chem* **2013**, *34* (19), 1672–1685. https://doi.org/10.1002/jcc.23317.
(25) Müller, M.; Hansen, A.; Grimme, S. ω B97X-3c: A Composite Range-Separated Hybrid DFT Method with a Molecule-Optimized Polarized Valence Double- ζ Basis Set. *J Chem Phys* **2023**, *158* (1). 10.1063/5.0133026.
(26) Neugebauer, H.; Pinski, P.; Grimme, S.; Neese, F.; Bursch, M. Assessment of DLPNO-MP2 Approximations in Double-Hybrid DFT. *J Chem Theory Comput* **2023**, *19* (21), 7695–7703. 10.1021/acs.jctc.3c00896.
